# Modality-specific sustained attention deficits in aphasia: task performance and lesion analysis

**DOI:** 10.1093/braincomms/fcaf479

**Published:** 2025-12-19

**Authors:** Svetlana V Kuptsova, Arianna N LaCroix, Oleg V Nikolsky, Marianna A Grigorian, Alexey G Petryshevskii, Maria V Ivanova

**Affiliations:** Department of Speech, Language, and Hearing Sciences, Purdue University, West Lafayette, IN 47907, USA; Department of Speech, Language, and Hearing Sciences, Purdue University, West Lafayette, IN 47907, USA; School of Information, University of Michigan, Ann Arbor, MI 48109, USA; Department of Clinical Psychology, Center for Speech Pathology and Neurorehabilitation, Moscow 109240, Russia; Department of Radiology, Center for Speech Pathology and Neurorehabilitation, Moscow 109240, Russia; Department of Psychology, University of California, Berkeley, CA 94720, USA

**Keywords:** aphasia, sustained attention, VLSM, modality specificity

## Abstract

People with aphasia experience cognitive deficits in addition to their well-documented language impairments, with attention being a frequently affected domain. While numerous studies highlight the impact of attention deficits on language abilities in people with aphasia, research examining the neural correlates of these deficits and the influence of stimulus modality on attention performance remains limited. In this cross-sectional study, in Experiment 1, we investigated sustained attention deficits in people with aphasia using four matched tasks that varied by modality (visual versus auditory) and linguistic content (verbal versus non-verbal). In Experiment 2, we further explored whether the neural resources supporting sustained attention differ by modality. Participants included 56 people with aphasia following left-hemisphere stroke (Experiments 1 and 2), 14 individuals after right-hemisphere stroke and 23 age-matched healthy controls (only Experiment 1). Participants completed four sustained attention tasks. Structural MRI scans were obtained for stroke participants, and neural correlates of attention were explored in people with aphasia. Behavioural analyses revealed that people with aphasia, as a group and across subtypes, performed significantly worse on auditory tasks with notably slower reaction times compared to healthy controls, indicating sustained attention deficits, particularly in the auditory modality. Compared to individuals with right-hemisphere stroke, people with aphasia made significantly more errors only on the auditory verbal task, which may reflect the combined impact of language impairment and attentional demands. No significant differences were observed between non-fluent and fluent aphasia subtypes, indicating comparable sustained attention deficits across these groups. However, participants with non-fluent aphasia demonstrated slower reaction times across nearly all tasks compared to healthy individuals, while those with fluent aphasia showed slower reaction times only in the auditory modality. Lesion-symptom mapping analysis did not reveal distinct brain–behaviour associations specific to the auditory modality. However, for the visual verbal task, poorer performance was associated with lesions in the inferior and middle frontal gyri and underlying white matter fasciculi (inferior fronto-occipital fasciculus, uncinate fasciculus and corpus callosum), regions implicated in written word comprehension. Taken together, these findings suggest that people with aphasia exhibit modality-specific sustained attention deficits, particularly in the auditory domain, likely reflecting impaired processing of auditory information.

## Teaser

Kuptsova *et al*. report that individuals with aphasia exhibit sustained attention deficits, particularly during auditory tasks. Their findings suggest a reduced ability to maintain auditory attention in aphasia, extending beyond language-specific impairments. Lesion analyses revealed that poorer visual verbal attention was associated with damage to left frontal and white matter regions.

## Graphical Abstract

**Graphical Abstract:**
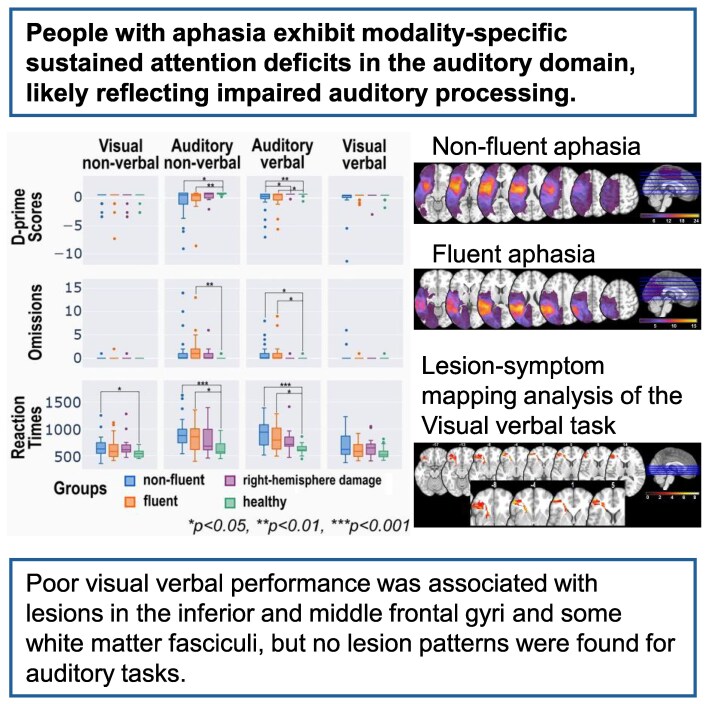

## Introduction

Aphasia is a language disorder caused by damage to the left hemisphere of the brain. People with aphasia (PWA) frequently exhibit impairments in both expressive and receptive language.^1,2^ However, aphasia is accompanied by impairments in other cognitive domains beyond language.^3-7^ Among cognitive functions, attention is frequently impaired.^5-20^ PWA with deficits in attention have poorer language outcomes than those with relatively preserved attention.^5,7,18,20-22^ Moreover, attention plays a critical role in language recovery, with some studies demonstrating that improvements in attention can facilitate language gains in PWA.^23-25^ Therefore, understanding how attention is affected in PWA is crucial for improving our understanding of aphasia recovery.

Attention can be divided into multiple components,^26^ with sustained attention or the ability to maintain focus on a specific task for an extended period, serving as a foundational process that supports more complex attentional operations. It enables individuals to maintain goal-directed behaviour and vigilance, particularly during repetitive or monotonous tasks. Sustained attention supports higher levels of attention, such as the ability to shift focus between two or more stimuli (alternating attention) as well as the ability to attend to relevant stimuli while ignoring others (selective attention).^20,27^ Sustained attention also supports language^5,18,21,28^ and is impaired in PWA compared to neurotypical controls,^13,18,29,30^ with one study demonstrating that ∼30% of left-hemisphere stroke survivors have sustained attention deficits,^31^ although the authors did not specify how many individuals with aphasia were included in the sample. A recent study suggests that sustained attention in PWA may be influenced by the nature of the stimuli, with linguistic tasks potentially placing greater attentional demands than non-linguistic ones.^30^ In this study, PWA and a matched-control group completed both linguistic and non-linguistic sustained attention tasks. The results showed that PWA made more errors on the linguistic tasks, but not on the non-linguistic task, compared to controls.^30^

Despite ample evidence indicating that PWA have impairments in sustained attention, studies investigating the lesion patterns associated with these deficits are sparse. Existing functional neuroimaging studies largely demonstrate that sustained attention is supported by a right-dominant network of frontal and parietal regions in neurotypical controls, including the bilateral pre-supplementary motor area, midcingulate cortex, inferior prefrontal cortex, premotor cortex, anterior insula and thalamus, as well as the right midlateral prefrontal cortex, temporoparietal junction, inferior parietal lobule, intraparietal sulcus and middle occipital gyrus.^32^ Studies exploring the neural substrate critical for sustained attention in PWA primarily focus on alertness, a key component of sustained attention that enhances and maintains readiness to process incoming information. While sustained attention enables prolonged focus on a task or stimulus, alertness refers to a general state of arousal and readiness to respond to stimuli.^33^ Alerting is therefore crucial for counteracting lapses in sustained attention, which in turn reduces the likelihood of missed information, improving overall task performance. Alertness has been associated with right lateralized frontoparietal regions in PWA.^34,35^ Rinne *et al*.^36^ further showed involvement of the thalamus in alerting after stroke, specifically in voxels projecting to the prefrontal cortex.

Overall, the current neural evidence strongly indicates that the right hemisphere plays a dominant role in sustained attention. Why, then, do PWA exhibit deficits in sustained attention? A meta-analysis by Langner and Eickhoff also identified the left inferior prefrontal cortex and premotor cortex as being involved in sustained attention, which is noteworthy since this region is frequently damaged in PWA. Additionally, research by Fonseca *et al*.^18^ found that individuals with non-fluent aphasia had greater sustained attention deficits than those with fluent aphasia. However, not all studies demonstrate differences in sustained attention across those with non-fluent and fluent aphasia,^21^ suggesting that more than the left prefrontal and premotor cortex may be contributing to sustained attention deficits in PWA. In support of this, models by Khomskaya^37^ and Luria^38^ classify attention by whether or not it employs modality-specific or non-specific neural resources. These models suggest that damage to modality-specific resources, such as the left auditory cortex, may also contribute to sustained attention deficits in aphasia. From this perspective, sustained attention to auditory information may depend in part on the integrity of left-hemisphere auditory regions, especially for linguistically rich input. Therefore, damage to modality-specific regions such as the left auditory cortex could contribute to sustained attention deficits in aphasia, particularly for auditory linguistic tasks.

This study aimed to comprehensively investigate sustained attention deficits in PWA. In Experiment 1, we assessed sustained attention using four matched tasks. Two tasks were visual, and two were auditory. Within each sensory modality, one task was verbal, and one was non-verbal. In Experiment 2, we investigated whether the neural resources supporting sustained attention differed by sensory modality and linguistic information using lesion-symptom mapping. We hypothesized that PWA would have poorer auditory than visual sustained attention and that these differences would be amplified for verbal tasks and a more severe aphasia diagnosis. We further expected that sustained attention deficits in PWA would be associated with damage to left frontal regions regardless of task modality and that damage to the left temporal lobe would be associated with poorer auditory sustained attention only. Last, if the left prefrontal and premotor cortices are critical to sustained attention, then we would expect that PWA with non-fluent aphasia would have poorer sustained attention than those with fluent aphasia. However, if the left auditory cortex plays a critical role in sustained attention, we would expect greater auditory sustained attention deficits in individuals with fluent aphasia, who typically have more extensive damage to posterior temporal regions, compared to those with non-fluent aphasia whose lesions are more often anterior.

## Experiment 1

### Materials and methods

#### Participants

We recruited 56 PWA (33 males) who had a single left-hemisphere stroke at least 3 months before testing (*M*  *=* 35.23 months post-onset, SD *=* 48.88). PWA ranged in age from 31 to 68 years (*M*  *=* 50.01, SD *=* 10.60), 53.6% had a vocational degree, and 46.4% had a university degree. PWA were pre-morbidly right-handed, were native speakers of Russian and had normal or corrected-to-normal vision and hearing, with no history of neurodegenerative disorders, epilepsy, other psychiatric disorders such as depression (as diagnosed by certified psychiatrists) or of alcohol or drug abuse. Aphasia types were classified according to Luria’s aphasia classification system^39,40^ by a speech-language pathologist and a neuropsychologist of the centre: 29 participants had non-fluent aphasia, 22 had fluent aphasia, and 5 had complex aphasia (i.e. they had deficits characteristic of both non-fluent and fluent subtypes). Here, we would like to emphasize that in Luria’s framework, the fluent/non-fluent distinction reflects not only speech output characteristics but also underlying neuropsychological mechanisms, such as the integrity of inner speech, auditory verbal memory, speech programming and other language-related processes. Thus, the assignment of umbrella terms ‘fluent’ and ‘non-fluent’ was not based on the scores of a single subtest targeting fluency but on the qualitative distinction between two major aphasic categories suggested by Benson and Ardila^41^ and Ardila^42^: anterior (non-fluent) and posterior (fluent). A more in-depth discussion of this classification approach and its relation to aphasia profiles can be found in our other publications.^3^ The severity of aphasia was assessed in a subset of 47 participants using the Assessment of Speech in Aphasia (see ‘Assessment of aphasia severity’ in this section for details).

We additionally recruited two control groups. The first control group represents neurotypical controls that do not have a history of stroke. The neurotypical control group was composed of 23 participants (10 males) who met the same inclusionary criteria as the aphasia group (i.e. they were right-handed, were native speakers of Russian and had normal or corrected-to-normal vision and hearing, with no history of neurological or psychiatric disorders or of alcohol or drug abuse). The neurotypical control group ranged in age from 31 to 70 years (*M*  *=* 53.09, SD *=* 10.37), 43.5% had a vocational degree, and 56.5% had a university degree.

The second control group were individuals who had right-hemisphere damage (RHD) due to stroke. The RHD control group included 14 individuals (10 males) who had a single right-hemisphere stroke at least 3 months prior to testing (*M*  *=* 31.71, SD *=* 20.04). The RHD group met the same inclusionary criteria as the aphasia group and ranged in age from 33 to 68 years (*M*  *=* 52.78, SD *=* 11.02), 57.1% had a vocational degree, and 42.9% had a university degree. This group did not present with aphasia, based on evaluations by a speech-language pathologist and a neuropsychologist at the centre. We included individuals with RHD due to substantial evidence linking RHD to sustained attention deficits.^32,43,44^ As they have attention deficits without language impairments, they serve as a useful control group for studying the specificity of sustained attention difficulties in PWA. Additionally, having a control group with brain damage but without aphasia who might exhibit attentional deficits different from PWA helps directly address a potential concern that attention deficits are simply due to diffuse effects of brain injury rather than damage to specific neural circuits.

**Figure 1:**
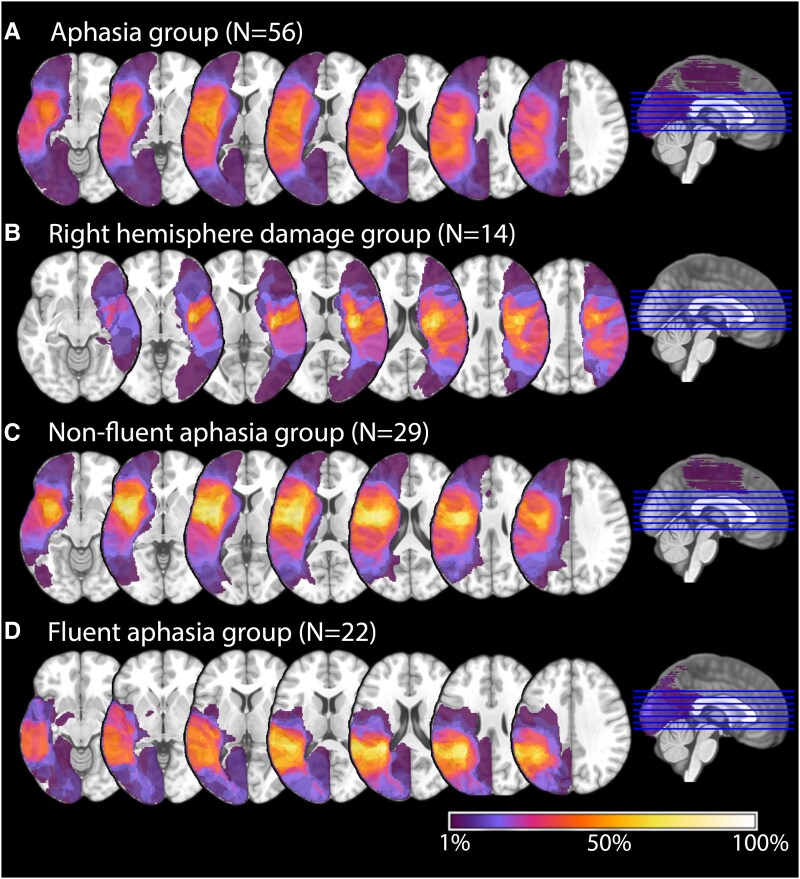
**Lesion overlay map for study groups.** Lesion overlay map showing distribution of lesions in (**A**) participants with aphasia following left-hemisphere damage (*N* = 56), with lesions in the left hemisphere in the inferior, middle and superior frontal regions; insula; precentral and postcentral gyri; superior, middle and inferior temporal regions, including the temporal pole; superior and inferior parietal regions; occipital region; basal ganglia; and deep midline brain structures; (**B**) participants with RHD (*N* = 14), with lesions in the right hemisphere in the inferior, middle and superior frontal regions; insula; precentral and postcentral gyri; superior, middle and inferior temporal regions, including the temporal pole; inferior and superior parietal regions; occipital region; basal ganglia; and deep midline brain structures; (**C**) participants with non-fluent aphasia (*N* = 29), with lesions in the inferior, middle and partially superior frontal regions; insula; precentral and postcentral gyri; superior and middle temporal regions, including the temporal pole; parietal regions; basal ganglia; and partially deep midline brain structures; and (**D**) participants with fluent aphasia (*N* = 22), with lesions in the superior, middle and inferior temporal regions, as well as parts of the temporal pole; parietal regions; precentral and postcentral gyri; occipital region; and parts of the putamen. The colour of each voxel represents the percentage of participants in the respective group who had a lesion in that voxel.

Participants with right- and left-hemisphere stroke were recruited from the Center for Speech Pathology and Neurorehabilitation in Moscow, Russia. The neurotypical control group was recruited using advertisements on social media networks and through relatives and acquaintances of staff and patients of the Center for Speech Pathology and Neurorehabilitation. This study was approved by the Ethics Committee of the Center for Speech Pathology and Neurorehabilitation. All participants gave written informed consent for participation in the study. Participants were not compensated for their time but participated due to an interest in research and desire to help others. Individual demographic data for all participants are presented in Supplementary Table 1, and lesion overlay maps for each post-stroke group are shown in Fig. 1.

#### Assessment of aphasia severity

Data on aphasia severity from the Assessment of Speech in Aphasia, which evaluates both production and comprehension,^45^ were available for 47 PWA. The production subtests include naming objects and actions, sentence construction, picture description and answering questions in a dialogue. The comprehension subtests include single-word auditory comprehension (both nouns and verbs), sentence comprehension, following commands and question comprehension in a dialogue. The maximum overall score is 300 for the whole battery. The higher the score, the fewer language impairments PWA have. For further details about this test, see Ivanova *et al*.^46^

#### Sustained attention tasks

Participants completed four tasks of sustained attention that were identical in design but differed regarding the sensory modality (auditory and visual) and type of stimuli (verbal and non-verbal). Within each sensory modality, one task was verbal, and one task was non-verbal. In each task, participants were instructed to focus on the stimuli and press the space bar as quickly as possible when a target stimulus appeared. Participants were instructed to not make a response if the stimulus presented was not the target (i.e. it was a foil). Each task consisted of 102 stimuli, including 21 target stimuli. Reaction time (RT) and accuracy were automatically recorded for each trial. Prior to the onset of the experimental task, participants were provided with written and verbal instructions and six practice trials, consisting of two targets and four foils, to ensure task comprehension. Participants had up to three opportunities to complete the practice trials. Only those who successfully completed the practice were included in the main experiment. All tasks were presented on a Lenovo ThinkPad laptop using E-Prime 2.0 and took 5.1 min to complete.

##### Visual sustained attention tasks

**Figure 2:**
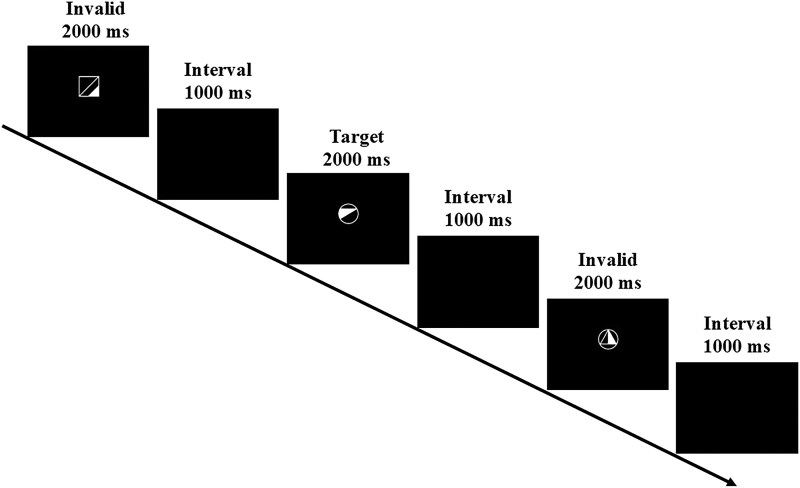
**Schematic of the visual sustained attention task procedure.** The target stimulus, labelled Target here, was used for half of the participants, while the other half’s target was the first stimulus here, labelled Invalid in the figure (i.e. the square).

Each trial began with the onset of a stimulus for 2000 ms, followed by a black screen for 1000 ms, resulting in a total interval of 3000 ms for each trial (Fig. 2). The stimuli for the ‘visual non-verbal sustained attention task’ included five white figures (geometric shapes) presented on a black background (Fig. 2). For this task, one half of the participants were assigned to the target stimulus labelled in Fig. 2, while the other half were presented with the first stimulus, labelled Foil 1, in Fig. 2 (i.e. the square). The stimuli for the ‘visual verbal sustained attention task’ included five high-frequency, four-letter, Russian words [glaz (eye), noch (night), stul (chair), krug (circle) and hleb (bread)] that were presented in white font on a black background. For this task, one half of the participants were assigned ‘stul’ as the target stimulus, while the other half received ‘glaz’ as the target stimulus.

##### Auditory sustained attention tasks

The ‘auditory non-verbal sustained attention task’ was identical to the visual task structure except each trial began with the onset of a stimulus for 500 ms, followed by no stimulus for 2500 ms, resulting in a total interval of 3000 ms for each trial. The stimuli included five different pure tones (250, 500, 750, 1000 and 2000 Hz) that were presented while participants fixated on a black screen. For this task, one half of the participants were assigned ‘250 Hz’ as the target stimulus, while the other half received ‘2000 Hz’ as the target stimulus.

The stimuli for ‘auditory verbal sustained attention task’ included five high-frequency spoken words having a length of four Russian letters [okno (window), reka (river), noga (leg), litso (face) and chasi (watch)]. Each word was ∼400 ms in length. A no-stimulus interval lasting for 2600 ms followed each word presentation, resulting in a total interval of 3000 ms for each trial. For this task, one half of the participants had the target stimulus ‘reka’, while the other half were presented with ‘litso’ as the target stimulus.

#### Statistical analysis of behavioural data

Statistical analysis was performed with SPSS Statistics for Windows, Version 22, and figures for behavioural data were created in Python 3.12.7 using library plotly 5.24.1. For each task, we excluded the first two stimuli, one of which was a target stimulus. This resulted in 100 stimuli being analysed, including 20 targets and 80 foils. Using the remaining 100 trials, we calculated *D*-prime (*D*′) for each task because it takes into account all errors (not just hits), making it more robust against response bias.^47^ To do this, we first *Z*-scored the hit rate and false positive rate and then subtracted the *Z*-scored false positive rate from the *Z*-scored hit rate. Higher *D*′ scores indicate better differentiation between signal and noise. While all trials were included in the *D*′ analyses, only trials associated with correct responses that had RTs within ±3 SD from each participant’s mean were included in the RT analysis. We also analysed omissions (i.e. missed targets) as a behavioural marker of sustained attention failure. Commission data (i.e. false alarms), reflecting inhibitory control, are mostly reported in the supplementary materials, as they were not central to our aims.

Prior to running any analyses, the data were first checked for normal distribution using the Kolmogorov–Smirnov test. Most dependent variables did not follow a normal distribution (*P* < 0.01), except for age (*P* > 0.05). To compare demographic variables across groups, a one-way ANOVA was used for age, Mann–Whitney and Kruskal–Wallis tests for time post-stroke, and a *χ*^2^ test for gender distribution. Because the main dependent variables (*D*′, omissions, commissions and RT) were not normally distributed, the non-parametric Kruskal–Wallis test was conducted for group comparisons. When a significant group effect was found, *post hoc* pairwise comparisons were performed and corrected for multiple comparisons using Dunn’s test with Bonferroni adjustment. When no overall group differences were detected, *post hoc* tests were not run. Spearman’s correlation analysis was performed to study the relationships between task performance and aphasia severity in all PWA and for fluent and non-fluent subgroups. Each analysis included 16 behavioural variables (*D*′, RT, omissions and commissions across four tasks) with *P*-values corrected using the Benjamini–Hochberg false discovery rate (FDR) method.

### Results

Of the 56 PWA initially recruited, 55 completed the sustained attention tasks involving figures, tones and written words, while 54 completed the auditory word sustained attention task. In the neurotypical control group, 23 participants completed the tasks involving tones, auditory words and written words, while 22 completed the figure sustained attention task. All 14 participants in the RHD group completed all sustained attention tasks. Missing data were due to technical issues during task administration and were not imputed in any analysis.

Since the target stimulus was counterbalanced across participants, we analysed whether differences existed between the two stimuli in each task to rule this out as a potential confounding factor. No differences were found between the two targets in any test (*P*  *>* 0.05), suggesting that the two targets in each test can be used interchangeably. Data are presented in Supplementary Table 2.

#### Group comparisons on demographics

There were no significant differences in age between the three main groups [*F*(2,90) = 0.868, *P* = 0.423], nor between the aphasia subgroups and the other groups [*F*(3,84) = 0.766, *P* = 0.662]. Similarly, there were no significant differences in months post-stroke between the PWA and RHD groups (U = 326.5, *Z* = −0.962, *P* = 0.336), nor between the aphasia subgroups and the RHD group (*H* = 1.153, *P* = 0.562). Finally, there were no significant differences in gender distribution across the three main groups [*χ*²(2, *N* = 93) = 2.047, *P* = 0.359], nor across all four groups [*χ*²(3, *N* = 88) = 2.420, *P* = 0.490]. These results indicate that the groups were comparable in terms of age, time post-stroke and gender and that these variables do not need to be included as covariates in subsequent analyses.

#### Behavioural results

##### Comparison of performance between PWA, RHD and neurotypical control groups

Means and SDs for omissions (missed targets), commissions (false alarms), *D*′ and RTs within each group and task are reported in Table 1.

**Table 1:** Descriptive statistics for omissions, commissions, *D*′ and RTs for each task and group

Performance measure		Visual non-verbal (figure)			Auditory non-verbal (tone)		
		PWA	RHD	Neurotypical	PWA	RHD	Neurotypical
Omissions	*M*(SD)	0.05 (0.29)	0.07 (0.27)	0.00 (0)	1.94 (3.58)	0.71 (1.64)	0.09 (0.29)
	Range	0–2	0–1	0–0	0–14	0–6	0–1
Commissions	*M*(SD)	0.27 (0.65)	0.21 (0.58)	0.23 (0.61)	3.73 (7.08)	1.57 (2.03)	0.35 (0.57)
	Range	0–3	0–2	0–2	0–30	0–5	0–2
*D*′	*M*(SD)	−0.074 (1.51)	−0.046 (1.32)	0.213 (0.98)	−0.426 (2.26)	0.369 (0.81)	0.796 (0.16)
	Range	−7.29–0.58	−3.35–0.58	0.20–0.89	−9.12–0.89	−2.02–0.89	0.20–0.89
RT	*M*(SD)	652.65 (190.16)	676.61 (219.55)	547.76 (77.92)	903.81 (292.32)	799.00 (256.81)	643.04 (132.43)
	Range	355.84–1251.80	470.40–1280.00	447.70–711.60	398.05–1626.00	458.25–1397.61	447.30–711.60

Performance measure		Visual verbal (written word)			Auditory verbal (audio word)		
		PWA	RHD	Neurotypical	PWA	RHD	Neurotypical
Omissions	*M*(SD)	0.20 (0.91)	0.00 (0)	0.04 (0.21)	1.18 (2.35)	0.07 (0.27)	0.04 (0.21)
	Range	0–6	0–0	0–1	0–10	0–1	0–1
Commissions	*M*(SD)	0.47 (1.37)	0.28 (1.06)	0.17 (0.39)	1.31 (3.23)	0.07 (0.27)	0.13 (0.46)
	Range	0–9	0–4	0–1	0–19	0–1	0–2
*D*′	*M*(SD)	−0.187 (2.13)	0.255 (0.93)	0.291 (0.54)	−0.434 (2.35)	0.639 (0.24)	0.630 (0.28)
	Range	−11.36–0.50	−2.96–0.50	−1.76–0.50	−11.99–0.70	−0.21–0.70	−0.60–0.70
RT	*M*(SD)	655.83 (196.30)	655.15 (193.21)	551.13 (106.04)	894.81 (272.17)	768.56 (231.28)	629.24 (87.47)
	Range	374.65–1229.42	404.05–1049.25	415.90–814.26	493.90–1740.30	470.42–1414.90	442.53–859.42

Omissions, missed targets (20 target trials per task); Commissions, false positive rate (80 foil trials per task); *D*′, subtracting the *Z*-scored false positive rate from the *Z*-scored hit rate; RT, reaction times for correct responses; PWA, participants with aphasia; RHD, participants with right-hemisphere damage; Neurotypical, neurotypical control participants.

Kruskal–Wallis tests revealed significant group differences in omissions and commissions for the auditory tasks between PWA and neurotypical control groups and also in omissions in the auditory verbal task between PWA and RHD groups. Data are reported in Table 2. Further, while PWA were slower across almost all tasks compared to neurotypical controls, they only performed worse (as indexed by *D*′) on the auditory tasks (tones and words) but not on the visual tasks (figures and written words) (Table 2). However, uncorrected *P*-values indicated significance for RTs across all tasks, although significance did not survive correction for multiple comparisons for the visual verbal task (Table 2). Compared to those with RHD, PWA performed worse on only the auditory verbal task (Table 2). RTs did not differ between PWA and those with RHD on any tasks (Table 2). Similarly, no significant differences (in *D*′ and RT) were observed between RHD and neurotypical controls.

**Table 2:** Kruskal–Wallis test results comparing PWA and control groups

Performance measure		Neurotypical versus PWA	RHD versus PWA	Neurotypical versus RHD
Omissions				
Visual non-verbal	*H*	1.38		
	*P*(adj.P)	0.502#		
Auditory non-verbal	*H*	18.272	9.053	−9.219
	*P*(adj.P)	0.001 (0.003)*	0.178 (0.533)	0.225 (0.676)
Auditory verbal	*H*	16.159	15.041	−1.118
	*P*(adj.P)	0.001 (0.004)*	0.013 (0.038)*	0.870 (1.000)
Visual verbal	*H*	1.230		
	*P*(adj.P)	0.541#		
Commissions				
Visual non-verbal	*H*	0.241		
	*P*(adj.P)	0.886#		
Auditory non-verbal	*H*	18.518	5.840	−12.679
	*P*(adj.P)	0.003 (0.008)*	0.434 (1.000)	0.134 (0.401)
Auditory verbal	*H*	13.295	14.270	0.975
	*P*(adj.P)	0.008 (0.024)*	0.018 (0.054)	0.886 (1.000)
Visual verbal	*H*	1.860		
	*P*(adj.P)	0.395#		
*D*′				
Visual non-verbal	*H*	0.695		
	*P*(adj.P)	0.706#		
Auditory non-verbal	*H*	−23.370	−9.040	14.331
	*P*(adj.P)	0.000 (0.001)*	0.239 (0.718)	0.100 (0.299)
Auditory verbal	*H*	−19.348.0	−19.792	−0.444
	*P*(adj.P)	0.000 (0.001)*	0.003 (0.008)*	0.953 (1.000)
Visual verbal	*H*	2.264		
	*P*(adj.P)	0.322#		
RT				
Visual non-verbal	*H*	16.036	−3.100	−19.136
	*P*(adj.P)	0.016 (0.048)*	0.695 (1.000)	0.034 (0.102)
Auditory non-verbal	*H*	28.848	9.181	−19.668
	*P*(adj.P)	0.000 (0.000)*	0.251 (0.752)	0.030 (0.089)
Auditory verbal	*H*	29.514	11.939	−17.575
	*P*(adj.P)	0.000 (0.000)*	0.132 (0.395)	0.050 (0.149)
Visual verbal	*H*	15.182	−0.747	−15.929
	*P*(adj.P)	0.022 (0.066)	0.926 (1.000)	0.078 (0.235)

Omissions, missed targets (20 target trials per task); Commissions, false positive rate (80 foil trials per task); *D*′, subtracting the *Z*-scored false positive rate from the *Z*-scored hit rate; RT, reaction times for correct responses; PWA, participants with aphasia; RHD, participants with right-hemisphere damage; Neurotypical, neurotypical control participants; #, multiple comparisons are not performed because the overall task does not show differences across samples; *, statistically significant difference between groups.

##### Comparison of *D*′, omissions and RTs between PWA subgroups and RHD and neurotypical control groups

**Figure 3:**
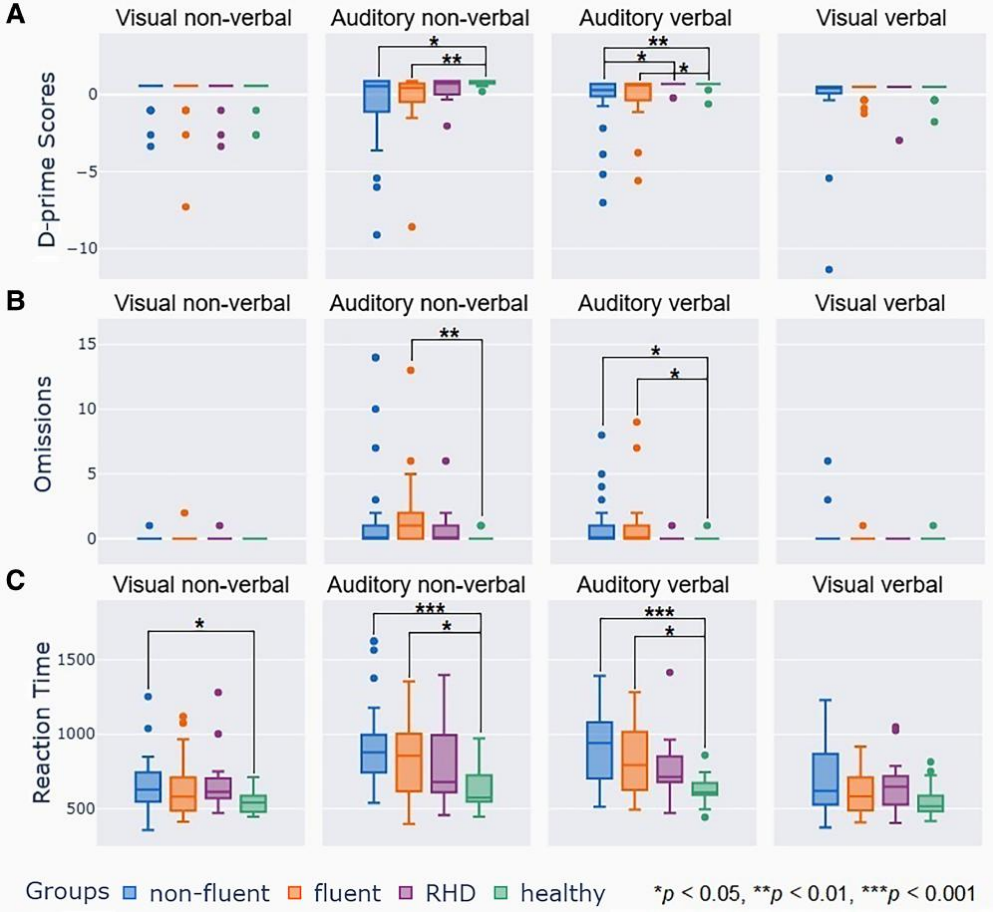
**Group differences in sustained attention performance.** Boxplots of Kruskal–Wallis test results (multiple comparisons) for *D*′, omissions and RTs, comparing fluent and non-fluent PWA to controls (healthy and RHD). The box represents the interquartile range, with the central line marking the median. The whiskers denote the largest/smallest values within 1.5 times the interquartile range above/below the 75th/25th percentile. Values falling outside of that range are shown as points. Each point represents one participant. The asterisk marks significant differences between groups according to the Kruskal–Wallis test. For corresponding statistical values, please see Table 3. (**A**) The *D*′ score is computed by subtracting the *Z*-scored false positive rate from the *Z*-scored hit rate. (**B**) Omissions represent missed targets. (**C**) RTs are measured in milliseconds. Sample sizes: non-fluent PWA: *N* = 28 (visual non-verbal and verbal, auditory non-verbal; *N* = 27 for auditory verbal); fluent PWA: *N* = 22; RHD: *N* = 14; and controls: *N* = 23 (visual verbal, auditory non-verbal and verbal; *N* = 22 for visual non-verbal).

An analysis of differences between the two PWA groups and controls revealed that individuals with non-fluent and fluent aphasia made significantly more errors than neurotypical controls on auditory tasks (both tones and words) but not on visual tasks, based on *D*′ (Table 3, Fig. 3). Omission analysis further showed that the non-fluent group had significantly more errors in the auditory verbal task (with a trend in the non-verbal task), while the fluent group showed significant errors in both auditory tasks. In terms of RT, participants with non-fluent aphasia were significantly slower than neurotypical controls on visual non-verbal and auditory tasks (both non-verbal and verbal), with a trend towards slower responses on the visual verbal task, while those with fluent aphasia showed slower RTs only in the auditory tasks (tones and words) (Table 3, Fig. 3). Based on *D*′, participants with non-fluent aphasia made more errors on the auditory verbal task compared to those with RHD, while the fluent group showed only a trend towards significance after correction, despite an uncorrected significant difference; omission trends were also observed in both aphasia groups for this task (Table 3). There were no differences in RT between RHD and non-fluent and fluent aphasia and RHD and neurotypical controls. We additionally compared performance between non-fluent and fluent aphasia and found no differences in *D*′, omissions or RTs between the two PWA groups (Table 3, Fig. 3). Commission data are reported in Supplementary Table 3.

**Table 3:** Kruskal–Wallis test results comparing fluent and non-fluent PWA to controls

Performance measure		Neurotypical versus non-fluent	Neurotypical versus fluent	Non-fluent versus fluent	Non-fluent versus RHD	Fluent versus RHD	Neurotypical versus RHD
Omissions							
Visual non-verbal	*H*	1.397					
	*P*(adj.P)	0.706#					
Auditory non-verbal	*H*	14.421	21.343	−6.922	5.607	12.529	−8.814
	*P*(adj.P)	0.015 (0.092)	0.001 (0.004)*	0.251 (1.000)	0.418 (1.000)	0.083 (0.498)	0.219 (1.000)
Auditory verbal	*H*	14.440	16.189	−1.748	13.378	15.127	−1.062
	*P*(adj.P)	0.007 (0.041)*	0.004 (0.023)*	0.746 (1.000)	0.031 (0.184)	0.019 (0.111)	0.868 (1.000)
Visual verbal	*H*	1.137					
	*P*(adj.P)	0.768#					
*D*′							
Visual non-verbal	*H*	0.426					
	*P*(adj.P)	0.935#					
Auditory non-verbal	*H*	−20.207	−24.034	3.826	−6.661	−10.487	13.547
	*P*(adj.P)	0.003 (0.018)*	0.001 (0.005)*	0.579 (1.000)	0.401 (1.000)	0.205 (1.000)	0.099 (0.593)
Auditory verbal	*H*	−18.587	−16.882	−1.705	−19.000	−17.295	−0.413
	*P*(adj.P)	0.001 (0.009)*	0.006 (0.035)*	0.773 (1.000)	0.005 (0.030)*	0.014 (0.083)	0.953 (1.000)
Visual verbal	*H*	1.866					
	*P*(adj.P)	0.601#					
RT							
Visual non-verbal	*H*	18.873	11.000	7.873	0.750	−7.123	−18.123
	*P*(adj.P)	0.008 (0.048)*	0.144 (0.864)	0.268 (1.000)	0.927 (1.000)	0.404 (1.000)	0.034 (0.203)
Auditory non-verbal	*H*	29.047	23.261	5.786	10.214	4.429	−18.832
	*P*(adj.P)	0.000 (0.000)*	0.002 (0.012)*	0.421 (1.000)	0.217 (1.000)	0.608 (1.000)	0.028 (0.167)
Auditory verbal	*H*	31.585	21.741	9.843	14.889	5.045	−16.696
	*P*(adj.P)	0.000 (0.000)*	0.004 (0.021)*	0.170 (1.000)	0.070 (0.421)	0.555 (1.000)	0.049 (0.291)
Visual verbal	*H*	17.806	10.686	7.120	2.393	−4.727	−15.413
	*P*(adj.P)	0.012 (0.073)	0.156 (0.936)	0.322 (1.000)	0.772 (1.000)	0.584 (1.000)	0.072 (0.431)

Omissions, missed targets (20 target trials per task); *D*′, subtracting the *Z*-scored false positive rate from the *Z*-scored hit rate; RT, reaction times for correct responses; PWA, participants with aphasia; RHD, participants with right-hemisphere damage; Neurotypical, neurotypical control participants; #, multiple comparisons are not performed because the overall task does not show differences across samples; *, statistically significant difference between groups.

##### Correlation between aphasia severity and performance on attention tasks

**Figure 4:**
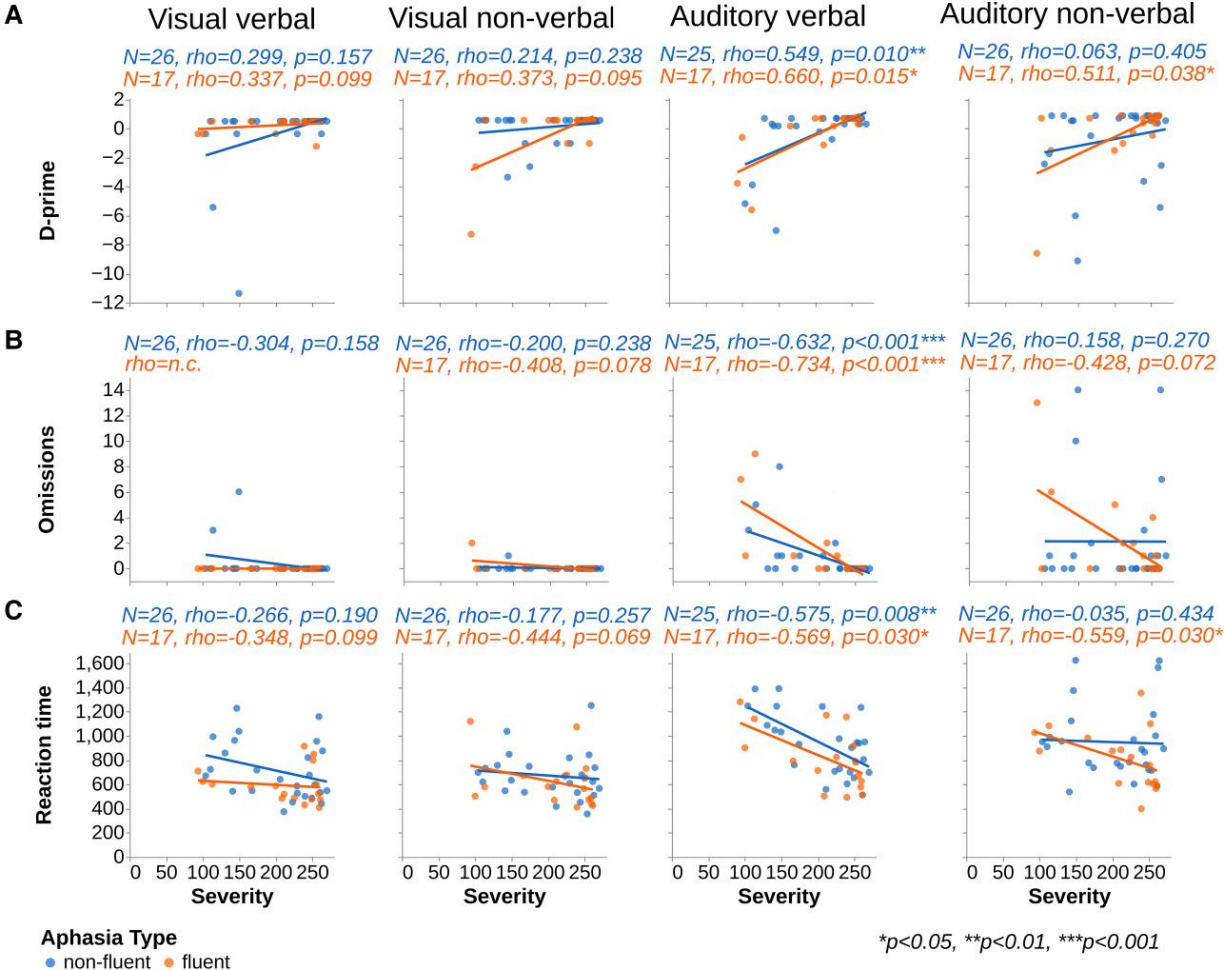
**Correlations between aphasia severity and sustained attention measures.** Spearman’s rho correlations between severity of aphasia and *D*′ (**A**), omissions (**B**) and RTs (**C**). Notes: not calculated (n.c.), correlation not calculated due to ceiling effect.

Although not a primary hypothesis, we conducted one-tailed Spearman’s correlations to examine the relationship between aphasia severity and task performance, based on the directional expectation that greater severity would be associated with poorer attention (i.e. lower *D*′, more omissions and slower RTs). This analysis was included to determine whether task performance was driven by general language severity. The whole group of PWA showed several moderate significant correlations between aphasia severity (as measured by the overall score on an aphasia battery) and *D*′, as well as omissions, for all the tasks except for the auditory non-verbal task. The strongest effects were observed for the auditory verbal task, where severity was strongly correlated with both *D*′ and omissions. For the RT, only RT from the auditory verbal task correlated with aphasia severity. Full correlation statistics are reported in Supplementary Table 4. To further explore what may be driving this relationship, we additionally analysed the relationship between performance on each task and severity separately within non-fluent and fluent aphasia groups. Within the non-fluent aphasia group, aphasia severity correlated with scores on *D*′, omissions and RTs for the auditory verbal task only. In contrast, within the fluent aphasia group, aphasia severity correlated with *D*′ and RTs for both the auditory tasks and with omissions for the auditory verbal task, showing a trend for the non-verbal task. The strongest effects in both groups were observed for omissions on the auditory verbal task. Results are reported in Supplementary Table 4 and presented graphically in Fig. 4.

## Experiment 2

### Materials and methods

#### Participants

**Figure 5:**
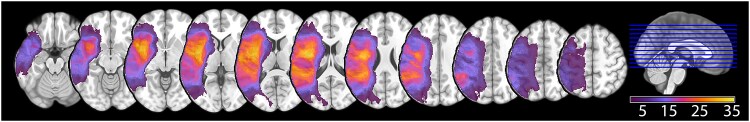
**Lesion overlay map of voxels included in the lesion-symptom mapping analysis for the PWA group.** The lesion overlay map shows the voxels included in the VLSM analysis (i.e. those voxels that at least five participants had lesioned, *N* = 56). This figure provides an anatomical summary and does not represent the results of a statistical test. The scale bar indicates the number of participants with overlapping lesions at each voxel.

The same 56 PWA from Experiment 1 completed the MRI portion of the study. The lesion overlay map is presented in Fig. 5. Neurotypical controls and individuals with RHD were not included in this experiment.

#### MRI data acquisition, pre-processing and lesion reconstruction

High-resolution T1-weighted, T2-weighted and fluid-attenuated inversion recovery (FLAIR) images were acquired using a 1.5 T Siemens Magnetom Avanto scanner. Scanning parameters differed slightly per participant, as sequences were performed based on each participant’s clinical needs. However, the majority of sequences were consistent across participants and included the following parameters: T1 [repetition time (TR) = 1900 ms, echo time (TE) = 3.37 ms, field of view (FoV) = 256 × 256 mm, slice thickness = 1 mm, 176 transverse slices]; T2 (TR = 5380 ms, TE = 94 ms, FoV = 512 × 512 mm, slice thickness = 5 mm, 22 transverse slices); and FLAIR (TR = 9000 ms, TE = 89 ms, FoV = 448 × 512 mm, slice thickness = 5 mm, 22 transverse slices). Scanning parameters that differed from these are reported in Supplementary Table 5.

The MRI data were pre-processed in SPM8 (http://www.fil.ion.ucl.ac.uk/spm). Prior to lesion reconstruction, the three images (T1, T2 and FLAIR) were manually reoriented to the anterior-posterior commissure (AC-PC) plane, the T1 was resliced to the MNI152 template with 1 mm^3^ resolution using fourth-degree B-spline transformation, and the T2 and FLAIR were co-registered and resliced to the new T1 using trilinear transformation.

The lesion masks were manually delineated using MRIcron (http://people.cas.sc.edu/rorden/mricron/index.html)^48^ and ITK-SNAP (Version 3.4.0-rc1, www.itksnap.org).^49^ To identify the lesion boundaries, we first delineated damaged tissue on the T1 and then used the T2 and FLAIR images to verify the lesion and make corrections, such as including adjoining white matter degeneration, gliosis and haemosiderin within the lesion mask. The T1 image and subsequently lesion masks were then normalized to the MNI152 template using a modified version of the unified segmentation/normalization algorithm implemented in SPM8 (‘Seg’ toolbox in the SPM8 distribution).^50^ This algorithm was customized to optimize normalization of deep white matter and ventricles by using an age-relevant template and by additionally incorporating a head model.^51^ Normalized lesions were once again inspected and visually compared to the lesions demarcated in native spaces. Cases of misalignment, such as lesion masks inside the ventricles or outside the meninges, as well as inconsistent extension to the cortical rim or the side of the ventricles, were manually corrected using segmentation tools in ITK-SNAP software. Automated Lesion Identification for SPM8 was used to generate lesion overlap maps (https://www.fil.ion.ucl.ac.uk/spm/ext/#toolboxes).

#### Voxel-based lesion-symptom mapping analysis

Voxel-based lesion-symptom mapping (VLSM) analyses were performed to establish the brain areas critical to sustained attention^52^ (Version 2.55, http://aphasialab.org/vlsm). In the VLSM analysis, a linear regression is estimated for each voxel, which compares behavioural performance (scores on *D*′, omissions, commissions and RT) in PWA with and without a lesion in that voxel. Additionally, participants’ age, months post-onset and lesion size were used as covariates in all the VLSM analyses to account for the possible influence of these variables. Only voxels that were lesioned in >10% (*N* = 5) of the patients were entered into the analysis (Fig. 4). Significance was set using cluster-based permutation testing with a voxel-wise threshold of *P* < 0.001 for *D*′ analysis and 0.005 for omission analysis, with permutations (*N* = 1000), and cluster size thresholding (*P* < 0.05). The brain regions associated with the significant voxels were identified in the Automated Anatomical Labeling (AAL) atlas and NatBrainLab atlas of white matter pathways^53^ in MRIcron using the Batch Descriptives function. A separate VLSM analysis was conducted for *D*′ scores and RT for each task, resulting in a total of eight separate VLSM analyses.

### Results

#### VLSM findings

**Figure 6:**
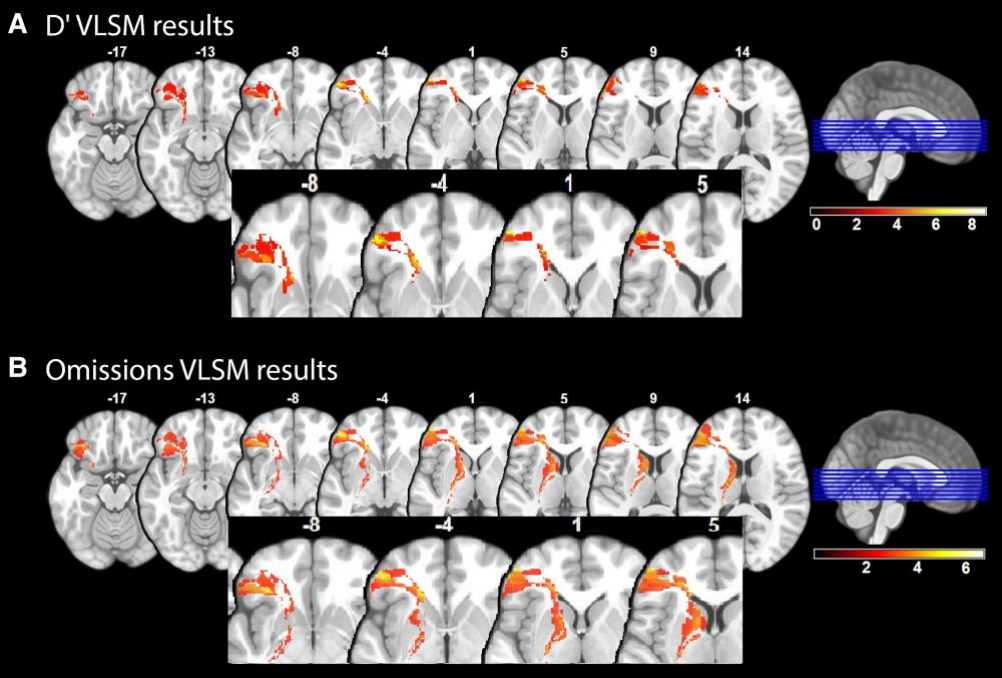
**VLSM results for the visual verbal sustained attention task.** The scale bar reflects *t*-statistic values. Brighter colours indicate increasing *t*-values and brain regions associated with poorer performance on this task when lesioned. (**A**) The VLSM analysis identified several clusters in cortical areas with *t*-values for *D*′ ranging from 3.26 to 8.51. Statistical analysis was performed using voxel-based linear regression with 1000 permutations, a voxel-wise threshold of *P* < 0.001 and cluster-level correction at *P* < 0.05. Sample size: *N* = 55. (**B**) The VLSM analysis identified several clusters in cortical areas with *t*-values for omissions from 2.68 to 6.71. Statistical analysis was performed using voxel-based linear regression with 1000 permutations, a voxel-wise threshold of *P* < 0.005 and cluster-level correction at *P* < 0.05. Sample size: *N* = 55.

We only obtained significant results in the VLSM analysis of *D*′ and omissions from the ‘visual verbal sustained attention task’ (Table 4, Fig. 6). The other three attention tasks did not yield any significant clusters in the VLSM analysis and yielded null maps.

**Table 4:** List of anatomical regions covered by the VLSM maps of the visual verbal sustained attention task (all regions are located in the left hemisphere)

	*D*′		Omissions	
	Number of voxels covered	Coordinates	Number of voxels covered	Coordinates
AAL atlas				
Inferior frontal gyrus, triangular part	2477	−55, 34, 15	5619	−55, 34, 15
Inferior frontal gyrus, orbital part	2968	−24, 24, −15	4436	−24, 24, −15
Middle frontal gyrus	1075	−45, 48, 1	1524	−49, 42, 18
Superior frontal gyrus, orbital part	181	−22, 13, −14	213	−22, 13, −14
Middle frontal gyrus, orbital part	945	−48, 49, −2	945	−46, 46, −2
Putamen	155	−18, 17, −10	1082	−14, 11, 1
Caudate nucleus	106	−17, 26, −4	1717	−15, 16, 20
Pallidum			1097	−25, −11, −1
Thalamus			203	−15, −19, 19
NatBrainLab atlas				
Corpus callosum	934	−16, 27, 13	1634	−16, 27, 13
Uncinate fasciculus	700	−17, 30, −1	840	−17, 30, −1
Inferior fronto-occipital fasciculus	428	−23, 36, −4	1031	−23, 36, −4
Cingulum			456	−13, 18, 22

The VLSM analysis of the visual verbal sustained attention task identified several similar clusters for both *D*′ and omissions in cortical areas associated with impaired performance, all located in the left hemisphere: middle frontal gyrus, superior frontal gyrus (orbital part), middle frontal gyrus (orbital part), inferior frontal gyrus (IFG, triangular part), IFG (orbital part), putamen and caudate nucleus. VLSM analysis also identified specific white matter pathways, including the corpus callosum, uncinate fasciculus (UF) and inferior fronto-occipital fasciculus (IFOF), where damage was associated with poorer performance on the visual verbal task.

## Discussion

The goal of the present study was to comprehensively quantify sustained attention deficits in PWA. To achieve this goal, we developed four analogous sustained attention tasks: visual non-verbal (figures), auditory non-verbal (tones), auditory verbal (audio words) and visual verbal (written words). We then analysed differences in performance on these tasks between PWA and the two control groups (neurotypical and RHD) and between PWA with non-fluent and fluent aphasia and how they each differed from the two control groups. In Experiment 2, we used lesion-symptom mapping to identify the neural substrates critical for each sustained attention task. Our results demonstrate that PWA performed worse on auditory sustained attention tasks than neurotypical controls; however, PWA only performed worse on the auditory verbal task compared to those with RHD. No differences in performance were observed between individuals with non-fluent and fluent aphasia. The VLSM analysis revealed significant findings only for the visual verbal sustained attention task. The implications of these findings are discussed below.

### Behavioural findings

In the current study, PWA exhibited deficits in auditory sustained attention tasks (both non-verbal and verbal) compared to neurotypical controls, but no differences emerged on visual sustained attention tasks, highlighting the auditory-specific nature of these deficits. This interpretation is supported by reduced *D*′ scores and increased omissions in auditory tasks among PWA, with the fluent group showing significant omissions in both verbal and non-verbal auditory tasks and the non-fluent group showing significance in the verbal task and a trend in the non-verbal task. Notably, all participants completed a training phase prior to each task, indicating that impaired performance on these attention tasks cannot be attributed solely to deficits in language comprehension. The auditory non-verbal task showed a selective impairment in PWA compared to neurotypical controls, whereas the visual non-verbal task did not, further suggesting that deficits extend beyond language processing and may reflect modality-specific limitations in sustained attention. Moreover, this auditory non-verbal deficit was associated with aphasia severity in the fluent group, suggesting involvement of the left auditory cortex in attentional processing of non-linguistic auditory input. These findings are consistent with prior research demonstrating impaired auditory sustained attention in PWA^5,13,14,21,29,54^ and align with one study showing greater impairments in auditory than visual attention in this population.^14^ Moreover, our results indicate no significant differences in sustained attention performance between individuals with fluent and non-fluent aphasia, a finding that corroborates Lee *et al*.^21^ but contrasts with Fonseca *et al*.,^18^ who reported worse performance in non-fluent aphasia. However, their study did not directly compare fluent and non-fluent groups; rather, each group was analysed separately against a control group (left-hemisphere stroke but no aphasia), limiting the interpretability of their conclusions. When directly comparing fluent and non-fluent aphasia, our results, along with those of Lee *et al*., suggest that sustained attention deficits are similar across different aphasia subtypes.

Both the non-fluent and fluent aphasia groups made more errors than the neurotypical control group on the auditory tasks, with individuals with non-fluent aphasia demonstrating significantly slower RTs across nearly all tasks, whereas those with fluent aphasia showed slower RTs only on auditory tasks (again, both verbal and non-verbal). However, while individuals with fluent aphasia did not significantly differ from RHD on any task, those with non-fluent aphasia demonstrated lower accuracy than RHD specifically on the auditory verbal task. Previous studies have reported that individuals with right frontal damage often experience severe impairments in sustained attention,^55,56^ while others have found no significant differences in sustained attention deficits between RHD and left-hemisphere damage groups.^56,57^ Our multiple-group comparisons revealed no significant differences between RHD and neurotypical controls or between RHD and the fluent aphasia group. However, we observed a trend towards significance in RTs between RHD and neurotypical controls and in auditory verbal task performance between RHD and fluent aphasia. Notably, individuals with non-fluent aphasia made significantly more errors on the auditory verbal task compared to both healthy controls and those with RHD, whereas those with fluent aphasia showed significant differences only from neurotypical controls and approached significance compared to RHD. These differences may indicate more severe sustained attention deficits in the non-fluent group.

Although the auditory verbal task might seem more challenging for individuals with fluent aphasia due to comprehension impairments characteristic of this type of aphasia, this task used simple, high-frequency words that participants with fluent aphasia were able to understand. Moreover, individuals who did not comprehend the task during the training session were not included in the study. Nonetheless, we acknowledge that task performance may reflect contributions from both sustained attention and modality-specific processing demands, including auditory and linguistic processing, and that the influence of these contributions might have varied across participants with aphasia. However, the observed group differences are unlikely to be explained solely by such individual variability or by outliers, as these were present across both auditory and visual tasks, primarily within the aphasia groups. Despite this variability, significant group-level differences emerged only in the auditory domain, both in the verbal and non-verbal tasks, with PWA showing more omissions and lower *D*′ scores than neurotypical controls. These findings indicate that while heterogeneity is not limited to auditory tasks, auditory processing imposes additional challenges for most individuals with aphasia. This aligns with Schumacher *et al*.,^35^ who reported that attention impairments are more pronounced in the auditory domain in individuals with aphasia and, yet, remain largely independent of language difficulties *per se*. In our study, the selective deficit observed in the non-fluent group, who typically have preserved single-word comprehension, combined with the similar performance of the fluent group and RHD controls, further suggests that language impairment alone does not fully explain the pattern of results. Instead, these findings point to increased attentional demands for processing verbal auditory information, particularly in individuals with anterior lesions, potentially exacerbating attention deficits in non-fluent aphasia.

### VLSM findings

A previous meta-analysis identified the left inferior prefrontal cortex and premotor cortex as key regions involved in sustained attention.^32^ Given this, it is notable that our VLSM analysis did not reveal significant clusters for either auditory sustained attention task, suggesting that additional factors may influence the relationship between lesion location and attentional performance. The lack of significant VLSM results, despite clear behavioural deficits in auditory sustained attention, suggests that these attentional processes can rely on a distributed neural network rather than being localized to a few specific regions. This distributed nature may limit the ability of lesion analysis in a relatively small sample to capture clear structure–function relationships. Additionally, functional reorganization following stroke may further obscure lesion-symptom mapping at the group level, as individuals with aphasia may recruit complementary brain areas, such as intact right-hemisphere regions or preserved sensory cortices, to compensate for direct damage to the attention network. Furthermore, the lack of significant clusters in auditory tasks may be attributed to high variability in performance across participants. No significant behavioural differences were observed between the two PWA groups, i.e. those with anterior lesions (non-fluent aphasia) versus those with posterior lesions (fluent aphasia), potentially reducing the sensitivity of lesion-deficit mapping. The absence of significant clusters in the visual non-verbal task may be due to the lack of participants with occipital lobe damage, which limits the ability to detect lesion-related effects in this modality.

The only task that showed significant associations between lesioned voxels and behavioural impairments was the ‘visual verbal sustained attention task’. Our findings indicate that poorer performance on this task was associated with damage to the inferior and middle frontal gyri. Meta-analytic research has demonstrated that the IFG plays a critical role in silent reading comprehension^58^ and is also crucial for integrating semantic and syntactic information, with its activation increasing in response to complex comprehension tasks, highlighting its role in managing linguistic workload.^58^ Furthermore, research has demonstrated a strong link between reading comprehension and working memory (WM) capacity, showing that individuals with lower WM capacity exhibit greater activation in the prefrontal cortex, likely reflecting increased executive control demands.^59^ Thus, these findings suggest that the visual verbal sustained attention task requires substantial engagement of prefrontal regions, possibly due to the need to employ executive control and linguistic processing resources.

Additionally, damage to specific white matter fasciculi was associated with poorer performance on the visual verbal sustained attention task. Due to their anatomical locations, both the IFOF and the UF support the ventral orthographic route.^60^ The IFOF connects the ventral occipital lobe to the orbitofrontal cortex and is crucial for orthographic processing. It facilitates the recognition of written words and their direct mapping to meaning, and disruptions in this tract have been linked to difficulties in word recognition and visual text processing.^60^ Consequently, participants with damage to the IFOF likely experienced challenges with word recognition, leading to poorer performance on tasks involving written words. The UF, another ventral tract, connects the anterior temporal lobe with the orbitofrontal cortex and facilitates semantic processing. It plays a significant role in integrating word meaning and context, which is essential for single-word reading comprehension. Damage to this tract has been associated with impaired semantic performance.^61^ PWA experience difficulties with understanding word meaning, and those who had damage to the UF experienced more difficulty on this task compared to those with an intact UF. The corpus callosum, which facilitates interhemispheric communication, is critical for integrating visual and linguistic information across the brain’s hemispheres.^61^ Damage to this tract was also observed in participants who had significant difficulties performing the written word task, further highlighting the importance of intact interhemispheric connectivity for efficient written word comprehension.

### Limitations of the current study

One of the main limitations of this study is the challenge in interpreting differences between fluent and non-fluent aphasia groups solely in terms of lesion location, given the variability and overlap in lesion patterns across participants. While individuals with non-fluent aphasia predominantly had frontal lobe damage, some also had temporal lobe involvement. Similarly, participants with fluent aphasia primarily had lesions in the temporal and posterior parietal lobes, with occasional extensions into the frontal lobes. Despite this overlap, the observed differences between the two groups can largely be attributed to anterior and posterior brain regions, as the majority of participants conformed to this expected pattern. However, we acknowledge that we cannot completely rule out the influence of posterior brain damage on sustained attention deficits in the non-fluent aphasia group.

A further limitation is that not all participants completed a standardized aphasia battery that would allow us to quantify their deficits, as such measures are not routinely used in Russian clinical practice.^62^ Aphasia types were classified using Luria’s qualitative framework, which lacks severity quantification, limiting severity analyses. Additionally, potential ceiling effects in hit rates, especially in the neurotypical controls and RHD groups, may have reduced sensitivity to detect subtle group differences, though our primary focus was on comparisons with the PWA group. We also acknowledge that the use of a binary fluent/non-fluent classification, although clinically common, may not fully capture individual differences in auditory comprehension. A more detailed grouping based on receptive language performance might better isolate the contribution of auditory processing to sustained attention.

An additional limitation of the current study is the relatively small sample size of the two aphasia groups. A larger sample for each aphasia subtype might have yielded more significant results both in the behavioural comparison between the two groups and in the VLSM analysis.

## Conclusion

We hypothesized that PWA would show greater deficits in auditory sustained attention than in visual sustained attention. Our findings confirmed impairments in auditory tasks, particularly for verbal stimuli. Aphasia severity was related to auditory sustained attention, with non-fluent aphasia showing deficits specific to verbal stimuli, whereas fluent aphasia exhibited more generalized auditory processing deficits. Although we initially expected sustained attention deficits to be linked to left frontal damage and manifest across all modalities, we found this association only in the time-based aspect of performance, with non-fluent PWA showing slower RTs across nearly all tasks. In contrast, accuracy deficits were primarily observed in the auditory domain, regardless of lesion location or aphasia type. Additionally, our findings did not support a dominant role of the frontal or temporal lobes in sustained attention, as both aphasia subgroups performed similarly. VLSM analysis also did not reveal distinct lesion-symptom associations specific to auditory modality or verbal/non-verbal tasks. Overall, these results suggest that sustained attention deficits in PWA are particularly pronounced in the auditory domain, likely reflecting a reduced capacity to maintain attention to auditory input.

## Supplementary Material

fcaf479_Supplementary_Data

## Data Availability

The data and materials related to this study that are not included in the article are available from the corresponding author upon reasonable request. The custom MATLAB scripts that were used for data analysis are included in the supplementary materials.
