# Supplementary material for: Modality-specific sustained attention deficits in aphasia: task performance and lesion analysis
Source: Brain Commun. 2025 Dec 19;7(6):fcaf479. doi: 10.1093/braincomms/fcaf479 (PMC12715772; doi:10.1093/braincomms/fcaf479)
Supplement: fcaf479_Supplementary_Data [file fcaf479_supplementary_data.docx]

**Supplementary Materials**

Supplementary Table 1. Individual characteristics of participants

| **#** | **Age** | **Sex** | **Months post-onset** | **Group** | **Type of aphasia** | **ASA** | **Lesion (in voxels)** | **D’ visual non-verbal** | **D’ auditory non-verbal** | **D’ auditory verbal** | **D’ visual verbal** | **RT visual non-verbal** | **RT tone** | **RT word** | **RT write** | **Omissions visual non-verbal** | **Omissions auditory non-verbal** | **Omissions auditory verbal** | **Omissions visual verbal** | **Commissions visual non-verbal** | **Commissions auditory non-verbal** | **Commissions auditory verbal** | **Commissions visual verbal** |
| --- | --- | --- | --- | --- | --- | --- | --- | --- | --- | --- | --- | --- | --- | --- | --- | --- | --- | --- | --- | --- | --- | --- | --- |
| 113 | 48 | m | 10 | PWA | comp | 234 | 77468 | 0.577 | -.0024 | 0.704 | 0.502 | 837.50 | 1599.17 | 923.21 | 730.20 | 0 | 8 | 0 | 0 | 0 | 1 | 0 | 0 |
| 162 | 47 | m | 32 | PWA | comp | 58 | 392871 | -4.221 | 0.711 | -11.99 | -8.688 | 602.80 | 698.10 | 1017.30 | 713.83 | 0 | 0 | 10 | 1 | 3 | 1 | 19 | 9 |
| 166 | 43 | f | 5 | PWA | comp |  | 123332 | -2.622 | -1.892 | -0.468 | 0.502 | 962.60 | 1350.61 | 1740.30 | 760.84 | 0 | 2 | 0 | 0 | 2 | 12 | 3 | 0 |
| 180 | 60 | m | 66 | PWA | comp | 248 | 129013 | -1.022 | 0.886 | 0.704 | -0.363 | 508.95 | 805.68 | 620.95 | 464.60 | 0 | 0 | 0 | 0 | 1 | 0 | 0 | 1 |
| 183 | 49 | f | 55 | PWA | comp | 198 | 159046 | 0.577 | 0.711 | 0.177 | 0.503 | 461.25 | 827.35 | 839.53 | 463.30 | 0 | 0 | 1 | 0 | 0 | 1 | 0 | 0 |
| 107 | 53 | m | 53 | PWA | non-fl | 270 | 64535 | 0.577 | 0.547 | 0.313 | 0.503 | 566.80 | 895.39 | 699.05 | 547.79 | 0 | 1 | 0 | 0 | 0 | 0 | 1 | 0 |
| 109 | 57 | m | 25 | PWA | non-fl | 206 | 27628 | 0.577 | 0.711 | -0.213 | 0.503 | 577.45 | 775.60 | 1243.89 | 641.55 | 0 | 0 | 1 | 0 | 0 | 1 | 1 | 0 |
| 112 | 37 | f | 20 | PWA | non-fl | 261 | 102196 | 0.577 | 0.886 | 0.313 | 0.503 | 623.10 | 587.80 | 512.60 | 534.00 | 0 | 0 | 0 | 0 | 0 | 0 | 1 | 0 |
| 116 | 56 | f | 37 | PWA | non-fl | 259 | 86724 | 0.577 | 0.372 | 0.704 | 0.503 | 1251.80 | 1001.50 | 1235.65 | 1160.42 | 0 | 1 | 0 | 0 | 0 | 1 | 0 | 0 |
| 126 | 57 | m | 6 | PWA | non-fl | 211 | 93603 | -1.022 | 0.886 | 0.704 | 0.503 | 417.45 | 749.05 | 559.05 | 374.65 | 0 | 0 | 0 | 0 | 1 | 0 | 0 | 0 |
| 152 | 46 | m | 12 | PWA | non-fl | 131 | 110559 | 0.577 | 0.886 | 0.704 | 0.503 | 757.90 | 956.40 | 1088.30 | 859.10 | 0 | 0 | 0 | 0 | 0 | 0 | 0 | 0 |
| 118 | 50 | f | 33 | PWA | non-fl | 264 | 42668 | 0.577 | -2.539 | 0.704 | 0.503 | 738.35 | 1622.85 | 950.53 | 876.65 | 0 | 7 | 0 | 0 | 0 | 6 | 0 | 0 |
| 123 | 45 | m | 48 | PWA | non-fl | 254 | 51095 | 0.577 | 0.711 | 0.704 | 0.503 | 355.84 | 715.30 | 946.05 | 596.95 | 0 | 0 | 0 | 0 | 0 | 1 | 0 | 0 |
| 124 | 44 | m | 7 | PWA | non-fl | 244 | 19670 | 0.577 | 0.886 | 0.704 | 0.503 | 452.00 | 866.05 | 700.60 | 821.50 | 0 | 0 | 0 | 0 | 0 | 0 | 0 | 0 |
| 127 | 46 | m | 5 | PWA | non-fl | 250 | 28976 | 0.577 | 0.547 | 0.704 | 0.503 | 653.05 | 722.63 | 654.50 | 486.80 | 0 | 1 | 0 | 0 | 0 | 0 | 0 | 0 |
| 139 | 64 | f | 3 | PWA | non-fl | 246 | 21983 | 0.577 | 0.886 | 0.704 | 0.503 | 680.05 | 762.35 | 904.40 | 676.60 | 0 | 0 | 0 | 0 | 0 | 0 | 0 | 0 |
| 144 | 54 | m | 22 | PWA | non-fl | 150 | 159386 | 0.577 | -9.116 | 0.177 | -11.36 | 634.20 | 1626.00 | 1033.44 | 1038.69 | 0 | 14 | 1 | 6 | 0 | 30 | 0 | 4 |
| 153 | 54 | m | 27 | PWA | non-fl | 229 | 110027 | 0.577 | 0.886 | 0.704 | 0.503 | 819.55 | 969.50 | 977.25 | 586.65 | 0 | 0 | 0 | 0 | 0 | 0 | 0 | 0 |
| 154 | 68 | f | 7 | PWA | non-fl | 111 | 55328 | 0.577 | -1.730 |  | 0.503 | 620.50 | 912.11 |  | 723.63 | 0 | 1 |  | 0 | 0 | 13 |  | 0 |
| 155 | 61 | m | 11 | PWA | non-fl | 230 | 152185 | -1.022 | 0.711 | 0.704 | -0.363 | 608.80 | 602.65 | 728.85 | 527.65 | 0 | 0 | 0 | 0 | 1 | 1 | 0 | 1 |
| 160 | 60 | m | 3 | PWA | non-fl | 114 | 104878 | 0.577 | 0.886 | -3.885 | -5.429 | 734.25 | 990.58 | 1389.40 | 993.88 | 0 | 0 | 5 | 3 | 0 | 0 | 5 | 2 |
| 161 | 34 | m | 26 | PWA | non-fl |  | 97433 | 0.577 | 0.536 | -2.185 | -0.363 | 548.50 | 555.35 | 686.69 | 454.47 | 0 | 0 | 4 | 0 | 0 | 2 | 2 | 1 |
| 175 | 55 | f | 77 | PWA | non-fl | 240 | 103263 | 0.577 | -3.634 | 0.313 | 0.503 | 532.10 | 889.81 | 604.80 | 501.65 | 0 | 3 | 0 | 0 | 0 | 20 | 1 | 0 |
| 104 | 39 | m | 17 | PWA | non-fl | 141 | 243323 | 0.577 | 0.536 | 0.313 | 0.503 | 547.35 | 537.95 | 1048.55 | 543.15 | 0 | 0 | 0 | 0 | 0 | 2 | 1 | 0 |
| 106 | 35 | m | 63 | PWA | non-fl | 168 | 225895 | -1.022 | -0.493 | 0.704 | 0.503 | 535.05 | 777.22 | 761.63 | 547.45 | 0 | 2 | 0 | 0 | 1 | 4 | 0 | 0 |
| 117 | 31 | m | 4 | PWA | non-fl | 223 | 98810 | 0.577 | 0.886 | -0.740 | 0.503 | 668.79 | 784.55 | 707.76 | 453.70 | 0 | 0 | 2 | 0 | 0 | 0 | 1 | 0 |
| 132 | 58 | f | 15 | PWA | non-fl | 104 | 193374 | 0.577 | -2.442 | -5.176 | -0.363 | 698.60 | 952.00 | 1248.76 | 670.65 | 0 | 0 | 3 | 0 | 0 | 19 | 11 | 1 |
| 137 | 40 | f | 27 | PWA | non-fl | 144 | 172347 | -3.354 | 0.547 | 0.177 | 0.503 | 1038.22 | 1124.33 | 1246.00 | 964.20 | 1 | 1 | 1 | 0 | 0 | 0 | 0 | 0 |
| 140 | 40 | m | 29 | PWA | non-fl | 256 | 76819 | 0.577 | 0.886 | 0.704 | 0.503 | 844.15 | 1177.45 | 940.55 | 956.25 | 0 | 0 | 0 | 0 | 0 | 0 | 0 | 0 |
| 143 | 39 | f | 19 | PWA | non-fl | 263 | 66982 | 0.577 | -5.439 | 0.704 | -0.363 | 510.47 | 1565.50 | 805.58 | 440.80 | 0 | 14 | 0 | 0 | 0 | 9 | 0 | 1 |
| 150 | 60 | f | 120 | PWA | non-fl |  | 294470 | 0.577 |  | -0.604 |  | 552.30 |  | 987.00 |  | 0 |  | 1 |  | 0 |  | 2 |  |
| 163 | 43 | m | 18 | PWA | non-fl | 147 | 155376 | 0.577 | -6.008 | -7.029 | -0.363 | 848.68 | 1375.90 | 1391.75 | 1229.42 | 0 | 10 | 8 | 0 | 0 | 20 | 9 | 1 |
| 181 | 59 | f | 317 | PWA | non-fl |  | 225099 |  | 0.536 |  | 0.503 |  | 773.11 |  | 526.15 | 0 | 0 |  | 0 |  | 2 |  | 0 |
| 182 | 57 | m | 107 | PWA | non-fl | 174 | 204119 | -2.622 | 0.886 | 0.177 | 0.503 | 749.25 | 738.00 | 932.21 | 718.15 | 0 | 0 | 1 | 0 | 2 | 0 | 0 | 0 |
| 105 | 43 | m | 3 | PWA | fluent | 261 | 2406 | 0.577 | 0.886 | 0.704 | 0.503 | 425.10 | 588.50 | 512.30 | 474.30 | 0 | 0 | 0 | 0 | 0 | 0 | 0 | 0 |
| 157 | 31 | f | 18 | PWA | fluent | 256 | 72930 | -1.022 | 0.372 | 0.704 | -1.229 | 474.25 | 604.16 | 670.90 | 599.80 | 0 | 1 | 0 | 0 | 1 | 1 | 0 | 2 |
| 110 | 48 | f | 5 | PWA | fluent |  | 23983 | 0.577 | 0.886 | 0.704 | 0.503 | 526.60 | 626.10 | 522.10 | 557.20 | 0 | 0 | 0 | 0 | 0 | 0 | 0 | 0 |
| 178 | 53 | f | 31 | PWA | fluent | 212 | 119286 | 0.577 | -1.018 | -1.131 | 0.503 | 623.68 | 885.65 | 1171.94 | 519.95 | 0 | 2 | 2 | 0 | 0 | 7 | 2 | 0 |
| 120 | 32 | m | 10 | PWA | fluent | 252 | 67952 | 0.577 | -0.471 | 0.704 | 0.503 | 731.21 | 1100.67 | 782.45 | 849.90 | 0 | 4 | 0 | 0 | 0 | 0 | 0 | 0 |
| 102 | 66 | m | 111 | PWA | fluent | 100 | 169302 | -2.622 | 0.711 | -0.604 | -0.363 | 502.10 | 875.74 | 901.67 | 623.70 | 0 | 0 | 1 | 0 | 2 | 1 | 2 | 1 |
| 108 | 34 | f | 17 | PWA | fluent | 251 | 45696 | 0.577 | 0.711 | 0.704 | 0.503 | 653.32 | 758.35 | 792.70 | 799.55 | 0 | 0 | 0 | 0 | 0 | 1 | 0 | 0 |
| 122 | 58 | m | 83 | PWA | fluent | 166 | 112971 | 0.577 | 0.711 | 0.177 | 0.503 | 670.90 | 983.84 | 792.78 | 580.60 | 0 | 0 | 1 | 0 | 0 | 1 | 0 | 0 |
| 125 | 59 | m | 15 | PWA | fluent | 209 | 98334 | 0.577 | 0.886 | 0.704 | 0.503 | 470.20 | 608.70 | 503.68 | 484.70 | 0 | 0 | 0 | 0 | 0 | 0 | 0 | 0 |
| 131 | 58 | m | 14 | PWA | fluent | 226 | 61903 | -1.022 | -0.318 | 0.177 | 0.503 | 677.89 | 824.88 | 825.17 | 488.32 | 0 | 2 | 1 | 0 | 1 | 3 | 0 | 0 |
| 134 | 56 | m | 15 | PWA | fluent |  | 151654 | 0.577 | -0.482 | 0.704 | 0.503 | 532.10 | 1001.82 | 814.10 | 576.05 | 0 | 3 | 0 | 0 | 0 | 2 | 0 | 0 |
| 147 | 68 | m | 7 | PWA | fluent |  | 138576 | 0.577 | -0.142 | -0.349 | 0.503 | 710.11 | 895.24 | 726.78 | 774.21 | 0 | 2 | 2 | 0 | 0 | 2 | 0 | 0 |
| 149 | 68 | f | 24 | PWA | fluent |  | 78265 | 0.577 | -0.318 | -1.131 | -0.897 | 733.89 | 1287.44 | 1014.72 | 669.63 | 0 | 2 | 2 | 1 | 0 | 3 | 2 | 0 |
| 151 | 50 | m | 124 | PWA | fluent | 94 | 281202 | -7.285 | -8.602 | -3.766 | -0.363 | 1120.50 | 1028.14 | 1281.85 | 709.65 | 2 | 13 | 7 | 0 | 0 | 29 | 2 | 1 |
| 156 | 43 | m | 16 | PWA | fluent | 200 | 74986 | 0.577 | -1.510 | 0.704 | -0.363 | 581.55 | 876.73 | 714.85 | 587.25 | 0 | 5 | 0 | 0 | 0 | 4 | 0 | 1 |
| 159 | 63 | f | 43 | PWA | fluent | 239 | 111681 | 0.577 | 0.372 | 0.704 | 0.503 | 1075.55 | 1355.42 | 1157.70 | 915.90 | 0 | 1 | 0 | 0 | 0 | 1 | 0 | 0 |
| 169 | 52 | f | 36 | PWA | fluent | 259 | 69346 | 0.577 | 0.711 | 0.313 | 0.503 | 487.35 | 621.85 | 576.58 | 523.20 | 0 | 0 | 0 | 0 | 0 | 1 | 1 | 0 |
| 174 | 36 | f | 27 | PWA | fluent | 249 | 58212 | 0.577 | 0.8861 | 0.704 | 0.503 | 564.40 | 617.74 | 918.80 | 475.75 | 0 | 0 | 0 | 0 | 0 | 0 | 0 | 0 |
| 176 | 66 | f | 50 | PWA | fluent | 260 | 40169 | 0.577 | 0.886 | 0.704 | 0.503 | 439.45 | 565.75 | 626.45 | 408.55 | 0 | 0 | 0 | 0 | 0 | 0 | 0 | 0 |
| 186 | 31 | m | 3 | PWA | fluent | 240 | 66689 | 0.577 | 0.711 | 0.704 | 0.503 | 412.05 | 398.05 | 493.90 | 430.80 | 0 | 0 | 0 | 0 | 0 | 1 | 0 | 0 |
| 130 | 50 | m | 4 | PWA | fluent | 113 | 53284 | 0.577 | -1.499 | -5.602 | 0.503 | 578.95 | 1084.07 | 1141.54 | 601.85 | 0 | 6 | 9 | 0 | 0 | 2 | 4 | 0 |
| 146 | 47 | f | 23 | PWA | fluent |  | 142458 | 0.577 | 0.547 | -0.214 | 0.503 | 966.75 | 832.79 | 1250.53 | 834.65 | 0 | 1 | 1 | 0 | 0 | 0 | 1 | 0 |
| 2 | 42 | m |  | NC |  |  |  | 0.577 | 0.711 | 0.704 | 0.503 | 484.11 | 539.45 | 659.40 | 438.45 | 0 | 0 | 0 | 0 | 0 | 1 | 0 | 0 |
| 21 | 48 | f |  | NC |  |  |  | 0.577 | 0.886 | 0.704 | -0.363 | 474.85 | 631.45 | 594.35 | 415.90 | 0 | 0 | 0 | 0 | 0 | 0 | 0 | 1 |
| 23 | 31 | m |  | NC |  |  |  | -2.622 | 0.886 | 0.704 | 0.503 | 478.85 | 971.80 | 859.42 | 814.26 | 0 | 0 | 0 | 0 | 2 | 0 | 0 | 0 |
| 24 | 53 | m |  | NC |  |  |  | 0.577 | 0.886 | 0.704 | 0.503 | 604.50 | 764.80 | 609.45 | 469.55 | 0 | 0 | 0 | 0 | 0 | 0 | 0 | 0 |
| 25 | 54 | f |  | NC |  |  |  | 0.577 | 0.886 | 0.704 | 0.503 | 491.11 | 569.80 | 567.89 | 669.60 | 0 | 0 | 0 | 0 | 0 | 0 | 0 | 0 |
| 26 | 40 | f |  | NC |  |  |  | 0.577 | 0.886 | 0.704 | 0.503 | 459.30 | 546.35 | 557.95 | 444.95 | 0 | 0 | 0 | 0 | 0 | 0 | 0 | 0 |
| 50 | 43 | f |  | NC |  |  |  | 0.577 | 0.711 | 0.313 | 0.503 | 534.50 | 529.90 | 603.65 | 480.25 | 0 | 0 | 0 | 0 | 0 | 1 | 1 | 0 |
| 51 | 60 | f |  | NC |  |  |  | 0.577 | 0.197 | 0.704 | 0.503 | 587.10 | 634.79 | 596.75 | 567.05 | 0 | 1 | 0 | 0 | 0 | 2 | 0 | 0 |
| 53 | 70 | f |  | NC |  |  |  | -2.622 | 0.886 | 0.704 | 0.503 | 612.00 | 693.26 | 632.25 | 498.21 | 0 | 0 | 0 | 0 | 2 | 0 | 0 | 0 |
| 54 | 68 | m |  | NC |  |  |  | 0.577 | 0.711 | 0.704 | 0.503 | 552.10 | 549.50 | 572.25 | 489.30 | 0 | 0 | 0 | 0 | 0 | 1 | 0 | 0 |
| 55 | 49 | m |  | NC |  |  |  | 0.577 | 0.886 | 0.704 | -0.363 | 530.35 | 533.40 | 611.00 | 577.45 | 0 | 0 | 0 | 0 | 0 | 0 | 0 | 1 |
| 56 | 63 | f |  | NC |  |  |  | 0.577 | 0.886 | 0.704 | 0.503 | 711.60 | 725.70 | 737.05 | 723.90 | 0 | 0 | 0 | 0 | 0 | 0 | 0 | 0 |
| 57 | 69 | m |  | NC |  |  |  | 0.577 | 0.886 | 0.704 | 0.503 | 705.75 | 775.30 | 746.00 | 591.26 | 0 | 0 | 0 | 0 | 0 | 0 | 0 | 0 |
| 58 | 50 | f |  | NC |  |  |  | 0.577 | 0.886 | 0.704 | 0.503 | 470.25 | 574.84 | 598.05 | 516.63 | 0 | 0 | 0 | 0 | 0 | 0 | 0 | 0 |
| 59 | 43 | f |  | NC |  |  |  | -1.022 | 0.886 | -0.604 | -1.763 | 581.25 | 919.95 | 734.21 | 751.00 | 0 | 0 | 1 | 1 | 1 | 0 | 2 | 1 |
| 60 | 53 | m |  | NC |  |  |  | 0.577 | 0.886 | 0.704 | 0.503 | 514.55 | 447.30 | 442.53 | 514.75 | 0 | 0 | 0 | 0 | 0 | 0 | 0 | 0 |
| 52 | 58 | m |  | NC |  |  |  | 0.577 | 0.886 | 0.704 | 0.503 | 466.20 | 562.10 | 496.21 | 431.05 | 0 | 0 | 0 | 0 | 0 | 0 | 0 | 0 |
| 61 | 49 | f |  | NC |  |  |  | 0.577 | 0.886 | 0.704 | 0.503 | 672.84 | 750.58 | 704.55 | 643.00 | 0 | 0 | 0 | 0 | 0 | 0 | 0 | 0 |
| 62 | 52 | m |  | NC |  |  |  | 0.577 | 0.886 | 0.704 | 0.503 | 547.75 | 527.65 | 677.85 | 522.40 | 0 | 0 | 0 | 0 | 0 | 0 | 0 | 0 |
| 63 | 63 | f |  | NC |  |  |  |  | 0.711 | 0.704 | 0.503 |  | 557.55 | 609.25 | 521.15 | 0 | 0 | 0 | 0 |  | 1 | 0 | 0 |
| 64 | 59 | f |  | NC |  |  |  | 0.577 | 0.711 | 0.704 | -0.363 | 551.65 | 553.90 | 629.90 | 508.70 | 0 | 0 | 0 | 0 | 0 | 1 | 0 | 1 |
| 65 | 63 | m |  | NC |  |  |  | 0.577 | 0.547 | 0.704 | 0.503 | 572.47 | 707.50 | 609.15 | 576.50 | 0 | 1 | 0 | 0 | 0 | 0 | 0 | 0 |
| 66 | 41 | f |  | NC |  |  |  | 0.577 | 0.711 | 0.704 | 0.503 | 447.70 | 723.16 | 623.35 | 510.65 | 0 | 0 | 0 | 0 | 0 | 1 | 0 | 0 |
| 119 | 37 | m | 23 | RHD |  |  | 150455 | 0.577 | 0.886 | 0.704 | 0.503 | 616.15 | 688.60 | 698.75 | 473.05 | 0 | 0 | 0 | 0 | 0 | 0 | 0 | 0 |
| 121 | 53 | m | 40 | RHD |  |  | 179691 | -2.622 | -0.318 | 0.704 | 0.503 | 749.90 | 974.00 | 730.45 | 1049.25 | 0 | 2 | 0 | 0 | 2 | 3 | 0 | 0 |
| 129 | 60 | m | 13 | RHD |  |  | 187673 | 0.577 | -0.154 | 0.704 | 0.503 | 1001.60 | 993.74 | 964.74 | 667.15 | 0 | 1 | 0 | 0 | 0 | 4 | 0 | 0 |
| 133 | 58 | m | 9 | RHD |  |  | 17828 | 0.577 | 0.010 | 0.704 | -2.960 | 616.05 | 648.95 | 695.00 | 648.10 | 0 | 0 | 0 | 0 | 0 | 5 | 0 | 4 |
| 135 | 42 | m | 11 | RHD |  |  | 100712 | 0.577 | 0.547 | 0.704 | 0.503 | 1280.00 | 1397.61 | 1414.90 | 1023.80 | 0 | 1 | 0 | 0 | 0 | 0 | 0 | 0 |
| 141 | 42 | m | 5 | RHD |  |  | 3532 | 0.577 | 0.886 | 0.704 | 0.503 | 611.40 | 883.20 | 678.58 | 787.15 | 0 | 0 | 0 | 0 | 0 | 0 | 0 | 0 |
| 142 | 46 | f | 35 | RHD |  |  | 158964 | -3.354 | 0.536 | 0.704 | 0.503 | 692.39 | 639.00 | 771.15 | 717.80 | 1 | 0 | 0 | 0 | 0 | 2 | 0 | 0 |
| 145 | 53 | m | 28 | RHD |  |  | 55967 | 0.577 | 0.886 | 0.704 | 0.503 | 604.60 | 598.35 | 573.50 | 528.16 | 0 | 0 | 0 | 0 | 0 | 0 | 0 | 0 |
| 165 | 33 | f | 53 | RHD |  |  | 13353 | 0.577 | 0.886 | 0.704 | 0.503 | 703.15 | 571.60 | 846.20 | 528.25 | 0 | 0 | 0 | 0 | 0 | 0 | 0 | 0 |
| 168 | 63 | f | 39 | RHD |  |  | 295549 | 0.577 | 0.886 | 0.704 | 0.503 | 570.80 | 612.00 | 517.60 | 453.21 | 0 | 0 | 0 | 0 | 0 | 0 | 0 | 0 |
| 173 | 61 | f | 18 | RHD |  |  | 3151 | -1.022 | 0.886 | 0.704 | 0.503 | 512.60 | 458.25 | 470.42 | 404.05 | 0 | 0 | 0 | 0 | 1 | 0 | 0 | 0 |
| 179 | 65 | f | 57 | RHD |  |  | 14090 | 0.577 | 0.361 | 0.704 | 0.503 | 573.00 | 995.25 | 850.20 | 646.05 | 0 | 0 | 0 | 0 | 0 | 3 | 0 | 0 |
| 184 | 58 | m | 41 | RHD |  |  | 222105 | 0.577 | -2.025 | 0.704 | 0.503 | 470.50 | 1054.36 | 865.42 | 598.90 | 0 | 6 | 0 | 0 | 0 | 5 | 0 | 0 |
| 185 | 68 | m | 72 | RHD |  |  | 16581 | 0.577 | 0.886 | -0.213 | 0.503 | 470.40 | 671.16 | 682.89 | 647.20 | 0 | 0 | 1 | 0 | 0 | 0 | 1 | 0 |

**Notes:** m – male; f – female; PWA – participants with aphasia; RHD – participants with right hemisphere damage; NC – neurotypical controls; comp – complex aphasia (non-fluent and fluent); non-fl – non-fluent aphasia; ASA – the Assessment of Speech in Aphasia.

Supplementary Table 2. Comparison of the two counterbalanced target stimuli within each task

| D’ visual non-verbal | *U =* 986.0, *z* = -.560, *p* = .576 |
| --- | --- |
| D’ auditory non-verbal | *U =* 858.5, *z* = -1.617, *p* = .106 |
| D’ auditory verbal | *U* = 937.0, *z* = -.931, *p* = .352 |
| D’ visual verbal | *U* = 981.5, *z* = -.841, *p* = .400 |
| Omissions visual non-verbal | *U =* 1012.5, *z* = -.577, *p* = .564 |
| Omissions auditory non-verbal | *U =* 863.5, *z* = -1.813, *p* = .070 |
| Omissions auditory verbal | *U* = 956.5, *z* = -.818, *p* = .413 |
| Omissions visual verbal | *U* = 990.0, *z* = -1.342, *p* = .180 |
| Commissions visual non-verbal | *U =* 1012.5, *z* = -.277, *p* = .782 |
| Commissions auditory non-verbal | *U =* 873.5, *z* = -1.539, *p* = .124 |
| Commissions auditory verbal | *U* = 1000.0, *z* = -.364, *p* = .715 |
| Commissions visual verbal | *U* = 1011.5, *z* = -.520, *p* = .603 |
| RTs visual non-verbal | *U* = 985.0, *z* = -.397, *p* = .691 |
| RTs auditory non-verbal | *U* = 967.0, *z* = -.707, *p* = .480 |
| RTs auditory verbal | *U* = 907.0, *z* = -1.016, *p* = .310 |
| RTs visual verbal | *U* = 980.0, *z* = -.605, *p* = .545 |

Supplementary Table 3. Kruskal-Wallis test results comparing commissions between fluent and non-fluent PWA and controls

| Performance measure |  | Neurotypical vs. non-fluent | Neurotypical vs. fluent | Non-fluent vs. fluent | Non-fluent vs. RHD | Fluent vs. RHD | Neurotypical vs. RHD |
| --- | --- | --- | --- | --- | --- | --- | --- |
| Commissions visual non-verbal | H | .007 | | | | | |
|  | *p* (*adj.p*) | 1.000# | | | | | |
| Commissions auditory non-verbal | H | 16.138 | 18.083 | -1.945 | 4.286 | 6.231 | -11.852 |
|  | *p* (*adj.p*) | .014(.087) | .010(.058) | .771(1.000) | .577(1.000) | .437(1.000) | .136(.816) |
| Commissions auditory verbal | H | 13.888 | 10.415 | 3.473 | 14.840 | 11.367 | .952 |
|  | *p* (*adj.p*) | .009(.055) | .063(.378) | .520(1.000) | .016(.099) | .077(.461) | .881(1.000) |
| Commissions visual verbal | H | 1.822 | | | | | |
|  | *p* (*adj.p*) | .610# | | | | | |

# - multiple comparisons are not performed because the overall task does not show differences across samples

Supplementary Table 4. Spearman's rho correlations between severity of aphasia and omissions, commissions, D’, and RTs.

|  | **PWA severity** | **Non-fluent severity** | **Fluent severity** |
| --- | --- | --- | --- |
| D’ visual non-verbal | *ρ* = .345, *p* = .023* | *ρ* = .214, *p* = .238 | *ρ* = .373, *p* = .095 |
| D’ auditory non-verbal | *ρ* = .185, *p* = .118 | *ρ* = .063, *p* = .405 | *ρ* = .511, *p* = .038* |
| D’ auditory verbal | *ρ* = .643, *p* < .001* | *ρ* = .549, *p* = .010* | *ρ* = .660, *p* = .015* |
| D’ visual verbal | *ρ* = .337, *p* = .022* | *ρ* = .299, *p* = .157 | *ρ* = .337, *p* = .099 |
| RTs visual non-verbal | *ρ* = -.214, *p* = .098 | *ρ* = -.177, *p* = .257 | *ρ* = -.444, *p* = .069 |
| RTs auditory non-verbal | *ρ* = -.181, *p* = .118 | *ρ* = -.035, *p* = .434 | *ρ* = -.559, *p* = .030* |
| RTs auditory verbal | *ρ* = -.522, *p* < .001* | *ρ* = -.575, *p* = .008* | *ρ* = -.569, *p* = .030* |
| RTs visual verbal | *ρ* = -.255, *p* = .061 | *ρ* = -.266, *p* = .190 | *ρ* = -.348, *p* = .099 |
| Omissions visual non-verbal | *ρ* = -.281, *p* = .045* | *ρ* = -.200, *p* = .238 | *ρ* = -.408, *p* = .078 |
| Omissions auditory non-verbal | *ρ* = -.011, *p* = .471 | *ρ* = .158, *p* = .270 | *ρ* = -.428, *p* = .072 |
| Omissions auditory verbal | *ρ* = -.718, *p* < .001* | *ρ* = -.632, *p* < .001* | *ρ* = -.734, *p* < .001* |
| Omissions visual verbal | *ρ* = -.330, *p* = .023* | *ρ* = -.304, *p* = .158 | n.c. |
| Commissions visual non-verbal | *ρ* = -.209, *p* = .098 | *ρ* = -.117, *p* = .326 | *ρ* = -.127, *p* = .313 |
| Commissions auditory non-verbal | *ρ* = -.326, *p* = .023* | *ρ* = -.204, *p* = .238 | *ρ* = -.615, *p* = .020* |
| Commissions auditory verbal | *ρ* = -.440, *p* = .004* | *ρ* = -.322, *p* = .158 | *ρ* = -.514, *p* = .038* |
| Commissions visual verbal | *ρ* = -.337, *p* = .023* | *ρ* = -.299, *p* = .158 | *ρ* = -.337, *p* = .099 |

Notes: *ρ* values reflect Spearman's rho correlation coefficients; *p*-values were corrected using the Benjamini-Hochberg FDR method; n.c. – not calculated, correlation not calculated due to ceiling effect.

Supplementary Table 5. Scanning parameters that differed from the presented in Method

| # | T1 | | | | T2 | | | | | FLAIR | | | | |
| --- | --- | --- | --- | --- | --- | --- | --- | --- | --- | --- | --- | --- | --- | --- |
|  | TR | TE | FoV | Number of slices | TR | TE | FoV | slice thickness | Number of slices | TR | TE | FoV | slice thickness | Number of slices |
| 102 |  |  |  |  | n/a | n/a | n/a | n/a | n/a |  |  |  |  |  |
| 104 |  |  |  |  | 4000 | 93 | 464x512 | 5 | 22 |  |  |  |  |  |
| 105 |  |  |  |  | 4000 | 93 | 464x512 | 5 | 22 |  |  |  |  |  |
| 106 |  |  |  |  | 4000 | 93 | 464x512 | 5 | 22 |  |  |  |  |  |
| 107 |  |  |  |  | 4000 | 93 | 464x512 | 5 | 22 |  |  |  |  |  |
| 108 |  |  |  |  | 4000 | 93 | 464x512 | 5 | 22 |  |  |  |  |  |
| 109 |  |  |  |  | 4000 | 93 | 464x512 | 5 | 22 |  |  |  |  |  |
| 110 | 8.62 | 4.74 | 512x512 |  |  |  |  | 4 | 24 |  | 90 |  | 4 | 24 |
| 112 |  |  |  |  | 5000 | 93 | 464x512 | 4 | 28 |  |  |  | 4 | 28 |
| 113 |  |  |  |  | 4000 | 93 | 464x512 | 5 | 22 |  |  |  |  |  |
| 116 |  |  |  |  | 4000 | 93 | 464x512 | 5 | 22 |  |  |  |  |  |
| 117 |  |  |  |  | 4000 | 93 | 464x512 | 5 | 22 |  |  |  |  |  |
| 119 |  |  |  |  | 4000 | 93 | 464x512 | 5 | 22 |  |  |  |  |  |
| 120 |  |  |  |  | 4000 | 93 | 464x512 | 5 | 22 |  |  |  |  |  |
| 121 |  |  |  |  | 4000 | 93 | 464x512 | 5 | 22 |  |  |  |  |  |
| 134 |  |  |  |  | 5000 | 93 | 464x512 | 4 | 28 |  |  |  | 4 | 28 |
| 135 |  |  |  |  | 5000 | 93 | 464x512 | 4 | 28 |  |  |  | 4 | 28 |
| 137 |  |  |  |  | 5000 | 93 | 464x512 | 4 | 28 |  |  |  | 4 | 28 |
| 140 |  |  |  |  | 5000 | 93 | 464x512 | 4 | 28 |  |  |  |  |  |
| 141 |  |  |  |  | n/a | n/a | n/a | n/a | n/a | n/a | n/a | n/a | n/a | n/a |
| 142 |  |  |  |  | 5000 | 93 | 464x512 | 4 | 28 |  |  |  | 4 | 28 |
| 146 |  |  |  |  | 5000 | 93 | 464x512 | 4 | 28 |  |  |  | 4 | 28 |
| 150 |  |  |  |  | 5000 | 93 | 464x512 | 4 | 28 |  |  |  | 4 | 28 |
| 161 |  |  |  |  | 5000 | 93 | 464x512 | 4 | 28 |  |  |  | 4 | 28 |
| 165 |  |  | 192x256 |  | n/a | n/a | n/a | n/a | n/a |  |  |  |  |  |
| 168 | 8.62 | 4.78 | 512x512 | 144 |  |  |  |  |  |  |  |  |  |  |
| 169 |  |  |  |  | 4000 | 93 | 464x512 | 5 | 22 |  |  |  |  |  |
| 179 |  |  |  |  | 5000 | 93 | 464x512 | 4 | 28 |  |  |  | 4 | 28 |
| 182 |  |  |  |  | 5000 | 93 | 464x512 | 5 | 22 |  |  |  |  |  |
| 185 |  | 2.91 |  |  | 4120 | 99 | 320x320 | 5 | 22 |  |  |  |  |  |
| 186 | 10.14 | 5.74 | 512x512 | 158 | 9716 | 110 | 672x672 | 3 | 44 |  |  |  |  |  |

**Source code used:**

**MATLAB**

For VLSM analysis:

vlsm('C:\Table.txt','C:\lesions',C:\output','vars',{'auditory_attention','age','months_poston_stroke','lesions'},'underlay','C:\MNI152_T1_1mm.nii','maskthresh',5, 'nperms', 1000)

For Calculating Benjamini Hochberg FDR for correlation analysis:

% Raw p-values from your correlation

p_raw = [data];

% Apply FDR correction (Benjamini-Hochberg)

p_fdr = mafdr(p_raw, 'BHFDR', true);

% Define significance threshold

alpha = 0.05;

is_significant = p_fdr < alpha;

% Create a table with a significance column

results_table = table((1:length(p_raw))', p_raw', p_fdr', is_significant', ...

'VariableNames', {'Index', 'Raw_p', 'FDR_corrected_p', 'Significant'});

% Display the table

disp(results_table)
